# Delivery of the *Pseudomonas aeruginosa* Phospholipase Effectors PldA and PldB in a VgrG- and H2-T6SS-Dependent Manner

**DOI:** 10.3389/fmicb.2019.01718

**Published:** 2019-07-31

**Authors:** Sarah Wettstadt, Thomas E. Wood, Selina Fecht, Alain Filloux

**Affiliations:** MRC Centre for Molecular Bacteriology and Infection, Department of Life Sciences, Imperial College London, London, United Kingdom

**Keywords:** type VI secretion system, bacterial toxin, phospholipase, VgrG, *Pseudomonas aeruginosa*

## Abstract

The bacterial pathogen *Pseudomonas aeruginosa* uses three type VI secretion systems (T6SSs) to drive a multitude of effector proteins into eukaryotic or prokaryotic target cells. The T6SS is a supramolecular nanomachine, involving a set of 13 core proteins, which resembles the contractile tail of bacteriophages and whose tip is considered as a puncturing device helping to cross membranes. Effectors can attach directly to the T6SS spike which is composed of a VgrG (valine-glycine-rich proteins) trimer, of which *P. aeruginosa* produces several. We have previously shown that the master regulator RsmA controls the expression of all three T6SS gene clusters (H1-, H2- and H3-T6SS) and a range of remote *vgrG* and effector genes. We also demonstrated that specific interactions between VgrGs and various T6SS effectors are prerequisite for effector delivery in a process we called “à la carte delivery.” Here, we provide an in-depth description on how the two H2-T6SS-dependent effectors PldA and PldB are delivered *via* their cognate VgrGs, VgrG4b and VgrG5, respectively. We show that specific recognition of the VgrG C terminus is required and effector specificity can be swapped by exchanging these C-terminal domains. Importantly, we established that effector recognition by a cognate VgrG is not always sufficient to achieve successful secretion, but it is crucial to provide effector stability. This study highlights the complexity of effector adaptation to the T6SS nanomachine and shows how the VgrG tip can possibly be manipulated to achieve effector delivery.

## Introduction

The type VI secretion system (T6SS) is a sophisticated protein secretion system that is found in about 25% of Gram-negative bacteria (Bingle et al., 2008). Resembling the contractile tail of bacteriophage (Kanamaru et al., 2002), the T6SS is used by bacteria to drive effector proteins directly into nearby cells, which can be of eukaryotic or prokaryotic nature (Basler et al., 2013). The T6SS is placed onto the bacterial cytoplasmic membrane *via* a membrane complex consisting of TssL, TssM and TssJ proteins (Durand et al., 2012, 2015). The membrane complex is connected to a so-called T6SS baseplate, sitting at the cytosolic side of the inner membrane and made of TssA, TssE, TssF, TssG, TssK, and VgrG (Brunet et al., 2015; Planamente et al., 2016). From the baseplate, the T6SS sheath is built and polymerizes into the cytoplasm. Composed of TssB and TssC subunits (Leiman et al., 2009), this helical contractile sheath envelops an inner tube, that consists of stacked hexamers of Hcp proteins (Brunet et al., 2014) which are topped by the VgrG spike (Renault et al., 2018). Contraction of the sheath toward the baseplate leads to extracellular release of the VgrG spike and the Hcp tube, where presence of Hcp in the supernatant fraction of bacterial cultures is a standard readout for T6SS activity, since it is a direct measure of a functional T6SS machinery (Pukatzki et al., 2006).

The spike atop the Hcp tube is composed of a trimer of VgrG proteins forming a rigid needle-like structure due to intertwining C-terminal hydrophobic β-sheets (Kanamaru et al., 2002). At the tip of the T6SS VgrG trimer sits a PAAR protein (Shneider et al., 2013) whose conical fold is thought to facilitate the puncturing of target membranes (Browning et al., 2012). One PAAR protein likely binds on top of one VgrG trimer in a way that it interacts with the last β-sheet derived from each VgrG monomer (Shneider et al., 2013).

*Pseudomonas aeruginosa* contains three T6SS clusters (H1-, H2- and H3-T6SS) each encoding all core T6SS components. The three clusters could be expressed simultaneously (Hachani et al., 2011; Allsopp et al., 2017) while each system delivers different sets of effector proteins into prokaryotic or eukaryotic cells (Russell et al., 2011; Hachani et al., 2014; Jiang et al., 2014; Whitney et al., 2014). Additionally, the *P. aeruginosa* genome contains multiple remote satellite islands, likely acquired *via* horizontal gene transfer, encoding different *vgrG, paar* and effector genes co-regulated with the core T6SS clusters (Jones et al., 2013; Allsopp et al., 2017). Effector genes are usually encoded together with immunity genes allowing the survival of the effector producing strain (Russell et al., 2011) as well as preventing T6SS-dependent intoxication by neighboring sibling cells (MacIntyre et al., 2010).

In some cases, the effector is a covalent extension of a structural component and thus called evolved VgrG-, PAAR-, or Hcp-effector (Ma et al., 2009, 2017; Whitney et al., 2015). If separated, a genetic link between an effector gene and a gene encoding a VgrG-, PAAR-, or Hcp-protein suggests a functional association (Hachani et al., 2014). Indeed, the delivery of many effectors was shown to be dependent on the nearby encoded VgrG, PAAR or Hcp component. In these cases, the effector protein specifically interacts non-covalently with a cognate Hcp hexamer, like Tse2 with Hcp1 in *P. aeruginosa* (Silverman et al., 2013); a PAAR protein, for example TseT with PAAR4 in *P. aeruginosa* (Burkinshaw et al., 2018); or the VgrG spike, as Tle1 with VgrG1 in *Escherichia coli* (Flaugnatti et al., 2015); a concept coined as “à la carte” effector delivery (Hachani et al., 2014). In some instances, the presence of so-called adaptor or chaperone proteins are required to connect effectors to the T6SS spike. These adaptor proteins can be of the DUF4123, DUF1795 or DUF2169 family. The DUF4123 proteins, denoted Tap1 (T6SS adaptor protein 1) from *Agrobacterium tumefaciens* and *Vibrio cholerae* were shown to be required for the recruitment of the effectors Tde1 and TseL, respectively, onto the cognate VgrG1 spikes (Liang et al., 2015; Unterweger et al., 2015; Bondage et al., 2016). DUF1795 proteins were termed Eag proteins and bind to the N-terminal half of PAAR effectors, chaperoning them in the producing cell before recruiting the PAAR effector to the T6SS tip (Diniz and Coulthurst, 2015; Whitney et al., 2015; Quentin et al., 2018). DUF2169 proteins seem to be less prevalent and their involvement in effector delivery has only been shown for Tde2 in *A. tumefaciens* (Bondage et al., 2016).

*Pseudomonas aeruginosa* encodes the two phospholipases PldA and PldB that were shown to be delivered into prey cells in a T6SS-dependent manner (Russell et al., 2013; Jiang et al., 2014; Boulant et al., 2018). Both effector genes are directly located downstream of two *vgrG* genes and no adaptor gene is encoded in their direct vicinity. This suggests that both effectors are recruited directly to the VgrG spike by a yet undefined mechanism. Here, we elucidated the effector delivery mechanisms of PldA and PldB *via* the T6SS of *P. aeruginosa* and describe an interesting new concept which shows that PldA delivery is not only dependent on one but two VgrG proteins.

## Materials and Methods

### Bacterial Strains and Growth Conditions

Bacterial strains used in this study are described in Table 1. *P. aeruginosa* strains were grown in tryptone soy broth (TSB) or LB supplemented with antibiotics where appropriate (streptomycin 2000 μg mL^–1^, spectinomycin 2000 μg mL^–1^, carbenicillin 100 μg mL^–1^, tetracycline 200 μg mL^–1^) at 37^∘^C with agitation. *E. coli* strains were grown in LB broth supplemented with antibiotics where appropriate (streptomycin 50 μg mL^–1^, kanamycin 50 μg mL^–1^, spectinomycin 100 μg mL^–1^, carbenicillin 50 μg mL^–1^, tetracycline 15 μg mL^–1^).

**TABLE 1:** Strains and plasmids used in this work.

**Strain or plasmid**	**Relevant characteristics^a^**	**Source/References**
Strain		
*E. coli*		
DH5α	F– Φ80*lac*ZΔM15 Δ(*lac*ZYA-*arg*F) U169 *rec*A1 *end*A1 *hsd*R17 (rK–, mK+) *pho*A *sup*E44 λ– *thi*-1 *gyr*A96 *rel*A1	Laboratory collection
CC118(λpir)	Host strain for pKNG101 replication; Δ(*ara-leu*) *araD*Δ*lacX74 galE galK-phoA20 thi-1 rpsE rpoB argE* (Am) *recA1 Rf^R^*λ*pir)*	Laboratory collection
*P. aeruginosa*		
PAO1	PAO1 wild type strain	Laboratory collection
PAO1Δ*rsmA*	Deletion in *rsmA* (*PA0905*)	Allsopp et al., 2017
PAO1::*pldA-bla_TEM–1_*	Insertion of *bla*_TEM–1_ before *pldA (PA3487)* STOP codon	Allsopp et al., 2017
PAO1::*pldB-bla_TEM–1_*	Insertion of *bla*_TEM–1_ before *pldB (PA5089)* STOP codon	Allsopp et al., 2017
PAO1ΔrsmAΔ*pldAtli5a*	Deletion in *rsmA*, *pldA-tli5a* (*PA3487-PA3488*)	This work
PAO1ΔrsmAΔ*pldAtli5a::lacZ*	Deletion in *rsmA, pldA-tli5a PA3487-PA3488*, chromosomal insertion of *lacZ* at *att* site	This work
PAO1ΔrsmAΔ*pldBtli5b_123_::lacZ*	Deletion in *rsmA*, *pldB-tli5b_123_* (*PA5089-PA5086*), chromosomal insertion of *lacZ* at *att* site	This work
PAO1ΔrsmAΔ*pldAtli5a* Δ*pldBtli5b_123_::lacZ*	Deletion in *rsmA, pldA-tli5a PA3487-PA3488, pldB-tli5b*_123_ *PA5089-PA5086*, chromosomal insertion of *lacZ* at *att* site	This work
PAO1Δ*rsmA*Δ*tssE2*	Deletions in *rsmA* and *tssE2* (*PA1657*)	This work
PAO1Δ*rsmA*Δ*tssK3*	Deletions in *rsmA* and *tssK3* (*PA2363*)	This work
PAO1Δ*rsmA*Δ*tssE2::pldA-bla_TEM–1_*	Deletions in *rsmA*, *tssE2* and insertion of *bla*_TEM–1_ before *pldA* STOP codon	Allsopp et al., 2017
PAO1Δ*rsmA*Δ*tssE2::pldB-bla_TEM–1_*	Deletions in *rsmA*, *tssE2* and insertion of *bla*_TEM–1_ before *pldB* STOP codon	This work
PAO1Δ*rsmA*Δ*tssK3::pldA-bla_TEM–1_*	Deletions in *rsmA*, *tssK3* and insertion of *bla*_TEM–1_ before *pldA* STOP codon	This work
PAO1Δ*rsmA*Δ*vgrG4b*	Deletions in *rsmA* and *vgrG4b* (*PA3486*)	Allsopp et al., 2017
PAO1Δ*rsmA*Δ*vgrG4b*Δ*pldBtli5b*_123_	Deletions in *rsmA, vgrG4b PA3486* and *pldB-tli5b*_123_ *PA5089-PA5086*	This work
PAO1Δ*rsmA*Δ*vgrG4b::pldA-bla_TEM–_*_1_	Deletions in *rsmA*, *vgrG4b* and insertion of *bla*_TEM–1_ before *pldA* STOP codon	This work
PAO1Δ*rsmA*Δ*vgrG4b::pldB-bla_TEM–1_*	Deletions in *rsmA*, *vgrG4b* and insertion of *bla*_TEM–1_ before *pldB* STOP codon	This work
PAO1Δ*rsmA::vgrG4b^618^-vgrG5^169^*	Deletion in *rsmA* and substitution of gene portion corresponding to *vgrG4b*^187^ with gene portion for *vgrG5*^169^	This work
PAO1Δ*rsmA::vgrG4b^618^-vgrG5^169^ ::pldA-bla_TEM–1_*	Deletion in *rsmA*, insertion of *bla*_TEM–1_ before *pldA* STOP codon, substitution of gene portion corresponding to *vgrG4b*^187^ with gene portion for *vgrG5*^169^	This work
PAO1Δ*rsmA*Δ*vgrG4b::vgrG5^623^-vgrG4b^187^*	Deletions in *rsmA*, *vgrG4b*, substitution of gene portion corresponding to *vgrG4b*^187^ with gene portion for *vgrG5*^169^	This work
PAO1Δ*rsmA*Δ*vgrG4b::vgrG5^623^-vgrG4b^187^::pldA-bla_TEM–1_*	Deletions in *rsmA* and *vgrG4b*, insertion of *bla*_TEM–1_ before *pldA* STOP codon, substitution of gene portion corresponding to *vgrG4b*^187^ with gene portion for *vgrG5*^169^	This work
PAO1Δ*rsmA*Δ*vgrG4b*Δ*tssE2::vgrG4b^618^-vgrG5^169^::pldA-bla_TEM–_*_1_	Deletions in *rsmA*, *vgrG4b, tssE2* and insertion of *bla*_TEM–1_ before *pldA* STOP codon and a substitution of gene portion corresponding to *vgrG4b*^187^ with gene portion for *vgrG5*^169^	This work
PAO1Δ*rsmA*Δ*vgrG4b*Δ*tssK3::vgrG4b^618^-vgrG5^169^::pldA-bla_TEM–1_*	Deletions in *rsmA*, *vgrG4b, tssK3* and an insertion of *bla*_TEM–1_ before *pldA* STOP codon and a substitution of gene portion corresponding to *vgrG4b*^187^ with gene portion for *vgrG5*^169^	This work
PAO1Δ*rsmA*Δ*vgrG4b*Δ*vgrG5*	Deletions in *rsmA*, *vgrG4b* and *vgrG5*	This work
PAO1Δ*rsmA*Δ*vgrG5*	Deletions in *rsmA* and *vgrG5 (PA5090)*	This work
PAO1Δ*rsmA*Δ*vgrG5::pldA-bla_TEM–1_*	Deletions in *rsmA* and *vgrG5* and insertion of *bla*_TEM–1_ before *pldA* STOP codon	This work
PAO1Δ*rsmA*Δ*vgrG5::pldB-bla_TEM–1_*	Deletions in *rsmA* and *vgrG5* and insertion of *bla*_TEM–1_ before *pldB* STOP codon	This work
PAO1Δ*rsmA*Δ*vgrG5::vgrG4b^618^-vgrG5^169^*	Deletions in *rsmA* and *vgrG5*, substitution of gene portion corresponding to *vgrG4b*^187^ with gene portion for *vgrG5*^169^	This work
PAO1Δ*rsmA*Δ*vgrG5::vgrG4b^618^-vgrG5^169^::pldB-bla_TEM–1_*	Deletions in *rsmA* and *vgrG5*, substitution of gene portion corresponding to *vgrG4b*^187^ with gene portion for *vgrG5*^169^ and insertion of *bla*_TEM–1_ before *pldB* STOP codon	This work
PAO1Δ*rsmA*Δ*TAA^vgrG4b^-ATG^pldA^*	Deletion in *rsmA*, deletion of STOP codon of *vgrG4b*, 16bp intergenic region and START codon of *pldA*	This work
PAO1Δ*rsmA*Δ*tssE2*Δ*TAA^vgrG4b^-ATG^pldA^*	Deletions in *rsmA* and *tssE2*, deletion of STOP codon of *vgrG4b*, 16bp intergenic region and START codon of *pldA*	This work
Plasmids		
pRK2013	Tra^+^Mob^+^, Km^R^	Laboratory collection
pCR2.1	TA cloning vector, Ap^R,^ Km^R^	Invitrogen
pCR-BluntII-TOPO	Blunt cloning vector, Zeo^R^, Km^R^	Invitrogen
pKNG101	Suicide vector, *sacB*, Str^R^	(39)
pKNG101-*vgrG4b*	pKNG101-*vgrG4b* deletion construct	Allsopp et al., 2017
pKNG101-*vgrG5*	pKNG101-*vgrG5* deletion construct	This work
pKNG101-*pldA-bla_TEM–1_*	pKNG101 deletion construct to fuse *bla*_TEM–1_ to *pldA* gene	This work
pKNG101-*pldB-bla_TEM–1_*	pKNG101 deletion construct to fuse *bla*_TEM–1_ to *pldB* gene	This work
pKNG101- *vgrG5^623^-vgrG4b^187^*	pKNG101 deletion construct to substitute final 493 bp of *vgrG5* with 564 bp from *vgrG4b*	This work
pKNG101- *vgrG4b^621^-vgrG5^169^*	pKNG101 deletion construct to substitute final 564 bp of vgrG4b with 493 bp from *vgrG5*	This work
pKNG1010 Δ*TAA^vgrG4b^-ATG^pldA^*	pKNG101 deletion construct to delete the STOP codon of *vgrG4b*, 16bp intergenic region and START codon of *pldA*	This work
pKNG101-*tssE2*	pKNG101-*tssE2* deletion construct	Allsopp et al., 2017
pKNG101-*tssK3*	pKNG101-*tssK3* deletion construct	Allsopp et al., 2017
pTrc200	Broad host range pVS1 derivative plasmid, *lacIq*, *pTrc* promoter, Sm^R^/Sp^R^	Schmidt-Eisenlohr et al., 1999; Gift from Erh-Min Lai
p *vgrG4b*	pTrc200 producing full length VgrG4b, Sm^R^/Sp^R^	This work
pBBR1-mcs4	Broad host range vector, with constitutive *PLAC* promoter, Ap^R^/Cb^R^	Kovach et al., 1995
p *vgrG5*-HA	pBBR1-MCS-4 encoding *vgrG5* with a C-terminal quadruple HA-tag, Ap^R^, Cb^R^	This work
p *vgrG5*^628^-HA	pBBR1-MCS-4 encoding *vgrG5*^628^ with a C-terminal quadruple HA-tag, Ap^R^, Cb^R^	This work
miniCTX::*lacZ*	Mini-CTX1 harboring the *lacZ* with constitutive promoter, Tc^R^	Becher and Schweizer, 2000

*^*a*^Ap^*R*^, ampicillin resistant; Str^*R*^, Streptomycin resistant; Km^*R*^, Kanamycin resistant; Tc^*R*^, Tetracycline resistant, Cb^*R*^ carbenicillin resistant, Sp^*R*^ spectinomycin resistant.*

### DNA Manipulation

DNA purification was performed using PureLink Genomic DNA mini kit (Life Technologies). Isolation of plasmid DNA was carried out using the QIAprep spin miniprep kit (Qiagen). Restriction endonucleases were used according to the manufacturer’s specifications (New England Biolabs or Roche). Oligonucleotides used are listed in Table 2 and were purchased from Sigma, United Kingdom. The genes or DNA fragments used for the construction of mutator plasmids and deletion mutants were amplified with KOD Hot Start DNA Polymerase (Novagen) as described by the manufacturer with the inclusion of 0.5 M betaine (Sigma). Colony PCR was performed with Taq polymerase (New England Biolabs). DNA sequencing was performed by GATC Biotech.

**TABLE 2:** Oligonucleotides used in this study.

**Deleted gene/ Oligonucleotide**	**Oligonucleotide sequence^*a*^**
*vgrG5*	
Up5′	ACAGCCATGTGCTCAACG
Up3′	GGTAGGCGTGGCGAACATTCACTGTCC
Down5′	ATGTTCGCCACGCCTACCGGCGCGACG
Down3′	CGCGATGAGGTTGAGGTT
*pldAtli5a*	
Up5′	GCGATCAAGATGCCGTTGAC
Up3′	TACTTCTTCCTTCTTCTGCAACATGGA TCAGTC
Down5′	CAGAAGAAGGAAGAAGTACTG CCCCGCC
Down3′	TTCTTCACCAGCATCTCGGT
*pldBtli5b*_123_	
Up5′	CCTGAAGCAGCCGGAACA
Up3′	CCGCAAACCTATCCTCTGCCTCCTCATCC
Down5′	CAGAGGATAGGTTTGCGGTTTGTACAGGT
Down3′	TAGTGATCGAGGCAGGCATG
*TAA^*vgrG4b*^-ATG^*pldA*^*	
Up5′	CAAGGACCAGAAGAAGCCCTACAA
Up3′	GGCTTCTTCTGGTCCTTGGCC
Gene fusions/ Oligonucleotide	
*pldA-bla_*TEM–1*_*	
Up5′	AAGCATCGCACAGCGGCCAGCCT
Up3′	AGGCTGGCCGCTGTGCGATG
Down5′	ATCGCACATGAAAAGGGTTTTGAT
Down3′	TACCTTCGCAGTTTGGCATG
*pldB-bla_*TEM–1*_*	
Up5′	CAAGATCGAGGCGCTCGAAG
Up3′	GTCAAAATCTTACGGTGGACGCGG CCAGCCTGGAAG
Down5′	GCATCGCTCAGTCCACCGTTACCAA TGCTTAATCAG
Down3′	ACAGAGCACGGCCCAAAGTC
*vgrG5^623^-vgrG4b^187^*	
Up5′	GGCGCCCTGGCACGGATCAAT
Up3′	GTGCCAGGGCGCCGTCGAGGGTG
Down5′	GCCAAGGACTGACAAGGATGAGC
Down3′	TCCTTGTCAGTCCTTGGCCAGT
*vgrG4b^621^-vgrG5^169^*	
Up5′	CGGCCCGCAGGTGATGATCAAC
Up3′	TCATCACCTGCGGGCCGCTGATGGC
Down5′	CGCCATGAGCCAAGGACTGAT
Down3′	CTCATGGCGTGGGCTCAT
Amplified gene/ Oligonucleotide	
*vgrG4b*	
5′	GCATACTCTAGAGTTGATCTGGTTGAGT TCCTTTTC
3′	GCATACGAGCTCTTTTCGAGAAACAGG GGACAG
*vgrG5-HA*	
5′	atgGTCGACGAATACGCCAGGAACGCAC
3′	tagGCTAGCGCCGCCGCTGTTGATCATCA
*vgrG5*^628^	
5′	atgGTCGACGAATACGCCAGGAACGCAC
3′	tagGCTAGCGCCGCCGCTGTTGATCATCA

*^*a*^Oligonucleotides are presented in the orientation 5′-3′.*

### Construction of *P. aeruginosa* Mutants

*Pseudomonas aeruginosa* deletion mutants were constructed as described previously (Vasseur et al., 2005) using the suicide plasmid pKNG101 (Herrero et al., 1990; Kaniga et al., 1991). Briefly, to create PAO1Δ*gene-of-interest* (*GOI*), 500-bp DNA fragments of the 5′ (up) and 3′ (down) ends of the target gene were obtained by PCR using PAO1 chromosomal DNA as a template with two pairs of oligonucleotides (Up5′/Up3′ and Down5′/Down3′) (Table 2). To create chimeric genes, splicing by overlap extension PCRs was performed. Briefly, approximately 500 bp upstream and downstream of the locus of interest was amplified using the internal primers 3′up and 5′down. Two additional primers, 3′down and 5′up, were used that contain complementary sequences to the opposing side of the splice junction and amplified to yield a fusion fragment. Thus, two subsequent overlap extension PCR steps were undertaken, employing an equimolar ratio of the upstream and downstream fragments as the DNA template. The gene fragments were cloned into pCR-BluntII-TOPO (Invitrogen), their sequences confirmed and sub-cloned into pKNG101 suicide vector. The pKNG-derivatives were maintained in the *E. coli* strain CC118λpir and mobilized into *P. aeruginosa* PAK using *E. coli* 1047 carrying the conjugative plasmid pRK2013 (Figurski and Helinski, 1979). Clones, in which double recombination events occurred, resulting in the deletion of *GOI* or fusion to *GOI*, were isolated using counterselection on sucrose plates as previously described (Vasseur et al., 2005). Gene deletion or fusion was verified by PCR using external primers and western blot analysis where appropriate.

### Secretion Assay

Secretion assays were performed similarly as previously described (Hachani et al., 2011). Bacterial suspension was diluted from overnight cultures in TSB to OD_600_ of 0.1 and grown at 25^∘^C to an OD_600_ of 4, unless otherwise stated. A bacterial culture sample adjusted to OD_600_ of 1 was harvested by centrifugation and served as the whole cell sample. Simultaneously, 13 mL of culture was centrifuged at 4 000 *g* for 20 min at 4^∘^C to separate the bacterial cells from culture supernatant. 10 ml of the supernatant was transferred into falcon tubes and centrifuged again; 7 mL of the uppermost supernatant was transferred into new tubes and centrifuged. To 1.8 mL supernatant fraction, we added 200 μl trichloroacetic acid to precipitate proteins overnight at 4^∘^C. The protein precipitate was centrifugated at 16 000 *g* for 30 min at 4^∘^C and washed with cold 90% (v/v) acetone before further centrifugation. After removing the supernatant, the washed pellet was air-dried for 30 min and resuspended in 1× Laemmli buffer to an OD_600_ equivalent of 20.

### Western Blot Analysis and SDS-PAGE

For SDS-PAGE analysis, cell extracts were loaded onto SDS polyacrylamide gels, migrated and transferred to a nitrocellulose membrane at 3 mA/cm^2^. Following transfer, membranes were incubated overnight in blocking buffer (5% milk powder, 0.1% Tween 20 in Tris–buffered saline, pH 8.0). Polyclonal antibodies against the C-terminal extension domain of VgrG4b (VgrG4b^C^) were used at a dilution of 1:1000, against Hcp2 at 1:1000 (Jones et al., 2013), against LasB 1:1000 (Gift from Romé Voulhoux). Monoclonal anti-Bla_TEM–1_ (BioLegend) and anti-HA antibodies (BioLegend) were used at a dilution of 1:5000. Monoclonal antibodies against the β subunit of RNA polymerase (RpoB, NeoClone) were used at 1:5000. Secondary antibodies conjugated to horseradish peroxidase were used at a dilution of 1:5000. Western blots were developed using Super-Signal West Pico Chemiluminescent Substrate (Pierce) and visualized on a LAS3000 Fuji Imager.

### Interbacterial Competition Assays

Interbacterial competition assays were conducted on solid media due to the contact-dependent killing of the T6SS (Hachani et al., 2013). *P. aeruginosa* prey strains contained the Mini-CTX-lacZ plasmid integrated at the *att* site, consequently giving rise to blue colonies on 5-bromo-4-chloro-3-indolyl-D-galactopyranoside (X-gal)-containing media. Overnight cultures in TSB were collected by centrifugation at 8 000 *g* for 3 min before washing twice in 1 ml sterile PBS and normalized to OD_600_ of 3.0. 100 μl of attacker and 100 μl prey strains for a ratio of 1:1 were mixed. This mixture was centrifuged at 8 000 *g* for 3 min and 100 μl supernatant was removed. 5 μl of each competition mix was spotted in duplicates onto LB-agar, the spots dried, and the Petri dish lids secured using parafilm M (Bemis). Competition plates were inverted and incubated at 25^∘^C for 24 h under H2-T6SS-inducive killing conditions (Allsopp et al., 2017).

The input competitions were serially diluted to 10^–7^, plated on selective media for both attacker and prey (LB agar with 100 μg mL^–1^ X-gal for blue/white differentiation) of *P. aeruginosa* prey/attacker and grown overnight at 37^∘^C to confirm the input ratios. Competition spots were gathered using 5 μl inoculation loops (VWR) and resuspended in 1 mL PBS. The competition output mixture was serially diluted to 10^–7^, plated on selective media and grown overnight at 37^∘^C similarly, to the input. Both attacker and prey colony forming units were enumerated on both input and output dilution plates. All competition assays were repeated three times unless otherwise stated and the mean colony forming unit (cfu) of surviving prey strains obtained from all experiments was plotted with the standard deviation.

## Results and Discussion

### PldA and PldB Are Delivered Into Prey Cells *via* Their Cognate VgrGs and in a H2-T6SS-Dependent Manner

**FIGURE 1:**
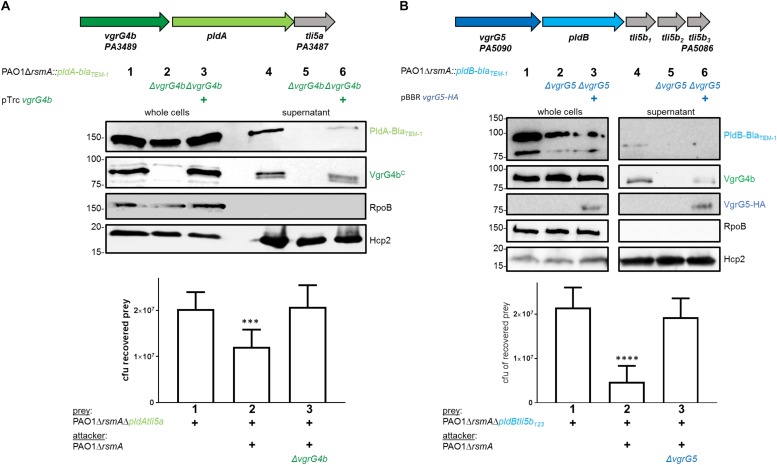
*P. aeruginosa* encodes two phospholipases delivered *via* their cognate VgrGs. **(A)** *vgrG4b* (PA3489, green) is encoded directly upstream of *pldA* (PA3488, bright green). **(B)** *vgrG5* (PA5090, blue) is encoded directly upstream of *pldB* (PA5089, cyan). Second panel: Representative figures of western blots from secretion assays using PAO1Δ*rsmA* expressing Bla_TEM–1_-tagged versions of PldA **(A)** or PldB **(B)**. The absence (Δ) of the native *vgrG4b* **(A)** or *vgrG5* **(B)** are indicated. For complementation experiments of the **(A)** *vgrG4b* and **(B)** *vgrG5* mutants, the introduction *in trans* of **(A)** *vgrG4b* (pTrc-*vgrG4b*) and **(B)** *vgrG5* (pBBR1-mcs5-*vgrG5-HA*) are marked with (+). Membranes in **(A)** and **(B)** were revealed, from top to bottom, using antibodies against Bla_TEM–1_, the C-terminal extension domain of VgrG4b (αVgrG4b^C^), the HA-tag, RpoB and Hcp2 as indicated on the right. In the bottom panel, bacterial competition outcomes are shown by plots of recovered cfu of prey strains susceptible to PldA delivery, PAO1Δ*rsmA*Δ*pldAtli5a::lacZ* **(A)**, or PldB delivery, PAO1Δ*rsmA*Δ*pldBtli5b_123_::lacZ* **(B)**, after contact with the attacker strain PAO1Δ*rsmA* encoding or lacking (Δ) the native *vgrG4b* **(A)** or *vgrG5* **(B)**. Spots were incubated for 24 h at 25 ^∘^C in a 1:1 ratio. One-Way ANOVA analysis with Dunnett’s multiple comparisons test was conducted on the data set obtained from recovered prey in the absence of an attacker strain with ^*⁣*⁣**^p < 0.0001.

The tandemly organized genes encoding the T6SS effector phospholipase PldA, also known as Tle5a (Russell et al., 2013), and the structural component VgrG4b are upregulated in an *rsmA* mutant and both proteins are secreted by the H2-T6SS in *P. aeruginosa* PAO1 (Supplementary Figure S1; Russell et al., 2013; Allsopp et al., 2017). Yet, this genetic link (Figure 1A, top panel) suggested PldA to be delivered as a VgrG4b-dependent effector, which we recently demonstrated by monitoring secretion of a chimeric fusion protein PldA-Bla_TEM–1_ (Allsopp et al., 2017). Here, we show that the lack of PldA secretion is solely due to the absence of VgrG4b since PldA release is restored by complementing the *vgrG4b* mutant (Figure 1A, middle panel, lane 6). PldA displays antibacterial activity (Russell et al., 2013) and we aimed at elucidating whether VgrG4b would facilitate PldA delivery into neighboring prey cells. We constructed prey strains lacking genes encoding PldA and its immunity Tli5a rendering them susceptible to PldA delivery. When in contact with the parental strain, the prey survival was challenged (Figure 1A, lower panel, lane 2), which was not the case when the attacker strain lacked *vgrG4b* (Figure 1A, lower panel, lane 3).

Interestingly, *P. aeruginosa* PAO1 produces a second T6SS phospholipase, PldB, also known as Tle5b (Russell et al., 2013), whose corresponding gene is found in a remote locus and downstream of a gene encoding VgrG5 (Figure 1B, top panel). To monitor PldB production and secretion using western blot analysis, we engineered a chimeric gene encoding a fusion between PldB and the β-lactamase, Bla_TEM–1_. Production of the PldB-Bla_TEM–1_ fusion protein is relieved in absence of RsmA (Supplementary Figure S2; Allsopp et al., 2017) and we assessed whether PldB delivery is mediated by VgrG5. Remarkably, PldB secretion was abrogated in absence of VgrG5 (Figure 1B, middle panel, lane 5), while PldB-mediated killing was abolished when using attacker strains lacking *vgrG5* (Figure 1B, bottom panel, lane 3). Complementation of PldB secretion with VgrG5 *in trans* could not be detected (Figure 1B, middle panel, lane 6) likely due to low abundance of PldB-Bla_TEM–1_ in complemented cells (lane 3). However, the used VgrG5 construct was functional since it was able to restore VgrG4b secretion, as will be discussed at a later point. Interestingly, secreted PldB-Bla_TEM–1_ was always detected as a smaller band (Figure 1B, lane 4) than in the whole cell fraction (lane 1). This is likely to be due to N-terminal processing of the effector as the detected Bla_TEM–1_ domain is fused to the C-terminus of PldB and can still be detected.

**FIGURE 2:**
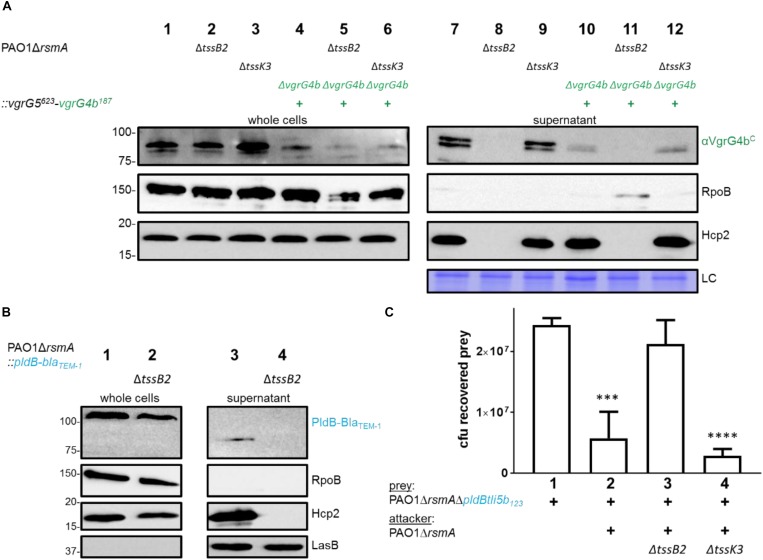
VgrG5 secretion and PldB delivery are H2-T6SS dependent. **(A)** Representative figure of a western blot from a secretion assay using PAO1Δ*rsmA.* Strains encode native *vgrG4b* or the *vgrG5^623^-vgrG4b^187^* chimera (+) as well as an active or inactive (Δ) H2-T6SS (Δ*tssB2*) or H3-T6SS (Δ*tssK3*). Antibodies used (from top to bottom) are against VgrG4b^C^, RpoB and Hcp2 as indicated on the right. A section from a Coomassie gel was included to serve as a loading control (LC) for the supernatant fraction. **(B)** Representative figure of a western blot from a secretion assay using PAO1Δ*rsmA* expressing a Bla_TEM–1_-tagged version of PldB. Strains produce an active or inactive (Δ*tssB2*) H2-T6SS. Antibodies used (from top to bottom) are against Bla_TEM–1_, RpoB, Hcp2 and LasB as indicated on the right. **(C)** Bacterial competition represented by plots of recovered cfu of prey strain PAO1Δ*rsmA*Δ*pldBtli5b_123_::lacZ* after contact with the attacker strain that encodes a non-functional H2-T6SS (Δ*tssB2*) or H3-T6SS (Δ*tssK3*). As a positive control for PldB-mediated killing, PAO1Δ*rsmA* was included. Spots were incubated for 24 h at 25 ^∘^C in a 1:1 ratio. One-Way ANOVA analysis with Dunnett’s multiple comparisons test was conducted on the data set obtained from recovered prey on its own with ^*⁣*⁣**^p < 0.0001.

A previous study suggested that PldB is delivered into prey cells by the H3-T6SS (Jiang et al., 2014). Here, not only do we show that PldB secretion is dependent on VgrG5, but we observed that VgrG5 secretion is more likely H2-T6SS-dependent. This statement is supported by our analysis of the secretion of a VgrG5^623^-VgrG4b^187^ chimera (first 623 amino acids of VgrG5, VgrG5^625^ and last 187 aa of VgrG4b, VgrG4b^187^, Supplementary Figure S3C). The VgrG5 portion of the chimera is the core gp27/gp5 domain while the C terminus has been replaced with the C terminus of VgrG4b, which we will describe in further sections. We reasoned that the N-terminal hub domains of the trimeric VgrG spike interact with the top Hcp hexamer of the Hcp tube likely mediating its affiliation to a specific T6SS (Renault et al., 2018). Hence, modifying the C-terminal domain of a VgrG tip protein would have no impact on its affiliation to a specific T6SS. The rationale for using this fusion is that in absence of a VgrG5 antibody we could monitor VgrG5 secretion by western blot analysis using an antibody against the C-terminal domain of VgrG4b. We used PAO1 wild type and mutant strains and showed that the VgrG5^623^-VgrG4b^187^ chimera is detected in the supernatant of H3-T6SS-inactive (Figure 2A, lane 12) but not of H2-T6SS-inactive mutants (Figure 2A, lane 11). We further reasoned that if VgrG5 is H2-T6SS-dependent then PldB might also be (Figure 2B). We assessed PldB secretion and showed that it was indeed abrogated when using H2-T6SS-inactive mutants (Figure 2B, lane 4). Finally, we performed bacterial competition assays (Figure 2C) and observed that PldB-mediated killing was only diminished from H2-T6SS inactive attackers (Figure 2C, lane 3) but not from those lacking an active H3-T6SS (Figure 2C, lane 4).

In all, we demonstrate that VgrG4b and VgrG5 do mediate delivery of their cognate effectors, PldA and PldB, respectively, which agrees with the “à la carte” delivery concept for genetically linked VgrG and cognate T6SS effector. We also showed that these two remote pairs, which are not genetically linked with other core T6SS genes, are H2-T6SS-dependent.

### The C-Terminal TTR-Like Domains of VgrG4b and VgrG5 Differ and Are Specific for Recognition of PldA and PldB

Recognition of T6SS effectors by a cognate VgrG could be direct or involve adaptor proteins facilitating the interaction. In *A. tumefaciens* it has been shown that the two VgrGs, VgrG1 and VgrG2, specifically bind and recognize their cognate effectors Tde1 and Tde2 (Bondage et al., 2016). In this case, Tde1 and Tde2 recognition involves two distinct adaptor proteins, Tap1, a DUF4123 protein, and Atu3641, a DUF2169 protein, respectively. The adaptor genes *tap1* and *atu3641* are genetically linked with the cognate effector genes *tde1* and *tde2*, respectively, and the adaptor proteins bind their cognate effectors to recruit them at the C-terminal regions of VgrG1 and VgrG2, respectively (Bondage et al., 2016).

**FIGURE 3:**
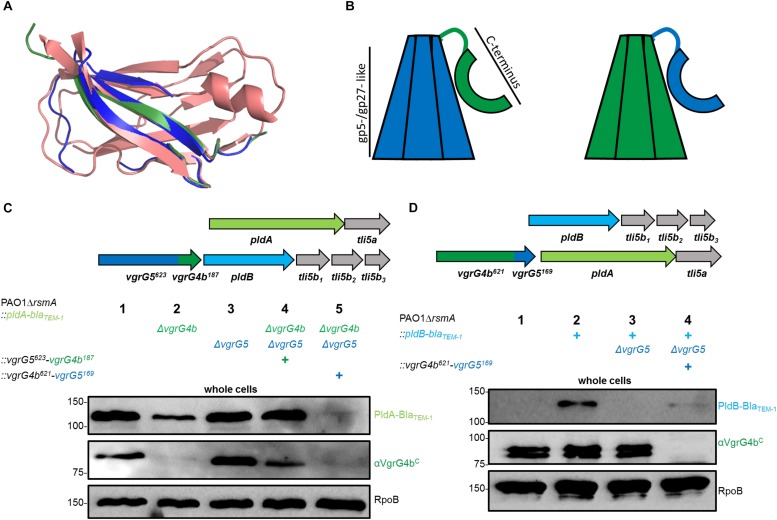
The C-terminal domain of a VgrG specifically stabilizes its cognate effector protein. **(A)** Structural models of the C termini of VgrG4b (green) and VgrG5 (blue) as compared to the transthyretin-like protein from *Salmonella dublin* (red, pdb: 2gpz). **(B)** Schematic of the here used chimerae between VgrG5 (blue) and VgrG4b (green). Highlighted are each the gp5-/gp27-like domains of VgrG4b and VgrG5 as well as the C-terminal domain consisting of the TTR-like domains (aa 648 – 770, see Supplementary Figure S3 for more details) as shown with an arc form. **(C,D)** Mutant strains carry Bla_TEM–1_-tagged version of PldA **(C)** or PldB **(D)** as well as the gene clusters as sketched in the top panels and using the color code as in Figure 1. **(C)** Strains express one of the following *vgrG* genes: native *vgrG5* (lane 2), native *vgrG4b* (lane 3), *vgrG5^623^-vgrG4b^187^* chimera (lane 4) or *vgrG4b^629^-vgrG5^169^* chimera (lane 5). Representative figures of western blots from whole cell fractions of PAO1Δ*rsmA* derivates as indicated by (+). **(D)** *vgrG4b^629^-vgrG5^169^* chimera replaces the native *vgrG4b* and the native *vgrG5* gene is deleted. Controls for native effector stability are shown with the parental strains in **(C)** lane 1 and **(D)** lane 2, while the negative controls lack the cognate *vgrG* locus in **(C)** lane 2 or in **(D)** lane 3. Antibodies used (top to bottom) are against Bla_TEM–1_, VgrG4b^C^ and RpoB as indicated on the right of each panel.

No adaptor genes are located in the vicinity of *pldA* or *pldB* suggesting that the corresponding effectors bind directly to their respective VgrG spikes. Using bioinformatic analysis, we identified transthyretin (TTR)-like folds within the C-terminal domains of both VgrG4b and VgrG5 (Figure 3A). Importantly, TTR-like domains within VgrG proteins have been shown to mediate binding of the cognate effector to the VgrG C terminus, as is the case in enteroaggregative *E. coli* and the VgrG1-dependent delivery of Tle1 (Flaugnatti et al., 2015).

The TTR-like domains of VgrG4b and VgrG5 share 25 % sequence identity, while their N-terminal gp5-/gp27-like domains share 69 % identity (Supplementary Figure S3A). From these observations, we hypothesized that specificity for PldA and PldB lies within the C-terminal domains of VgrG4b and VgrG5, respectively. To assess this concept, we swapped the C-terminal domains between VgrG4b (the C-terminal 187 amino acids) and VgrG5 (the C-terminal 169 amino acids) as depicted in Supplementary Figures S3B,C leading to chimeric VgrG proteins (Figure 3B). For VgrG protein detection we used an antibody that specifically recognizes the C terminus of VgrG4b as shown already in Figure 2A. As such, from the two chimeric VgrGs, that we engineered, the antibody could detect the VgrG5^623^-VgrG4b^187^ chimera (Figure 3C, lane 4), but not the VgrG4b^621^-VgrG5^169^ chimera (Figure 3D, lane 4). We also used strains that produce Bla_TEM–1_-tagged versions of PldA or PldB (Figures 3C,D, upper panels) so that production of these T6SS effectors could be followed with an antibody directed against the Bla_TEM–1_ portion of the chimeric proteins. We performed western blot analysis of whole cell lysates derived from various strains and observed that in absence of VgrG4b, there is a consistent decrease in the amount of the cognate effector PldA (Figure 3C, lane 2). A stable PldA protein is only seen in presence of VgrG4b (Figure 3C, lanes 1 and 3) or its C-terminal domain (Figure 3C, lane 4). Instead, the sole presence of VgrG5 (Figure 3C, lane 2) or its C-terminal domain (Figure 3C, lane 5) had no stabilizing effect on PldA. On the other hand, the absence of VgrG5 has an even more drastic impact on the abundance of its cognate effector PldB (Figure 3D, lane 3). Interestingly, PldB stability was partly restored when the C terminus of the cognate VgrG5 was expressed as part of a VgrG4b chimera (Figure 3D, lane 4), which would suggest that the cognate VgrG C terminus is sufficient to help stabilizing the effector.

Here, it is interesting to observe that stability of the T6SS effectors PldA and PldB depends on the C-terminal domains of their cognate VgrGs. On multiple occasions, it has been experimentally validated that T6SS effectors require the presence of cognate T6SS components for their stability. Tse2 from *P. aeruginosa* was shown to be degraded in absence of its receptor Hcp1 (Silverman et al., 2013), while purification of the effector Tde1 from *A. tumefaciens* led to higher yields in presence of its adaptor protein Tap1 (Ma et al., 2014). Furthermore, substrates from other bacterial secretion systems require the presence of dedicated chaperone proteins for stability. For example, the T3SS substrate YopE from *Y. pseudotuberculosis* (Birtalan et al., 2002) as well as the T4SS substrate VirE2 from *A. tumefaciens* (Zhao et al., 2001; Sutherland et al., 2012) require chaperones to connect to the cognate secretion machinery. For PldA and PldB, the C-terminal domains of their cognate VgrGs likely act as chaperone domains.

The mechanism involving specific binding and stabilization of a toxic protein by a secretion component prior to its export likely represents a survival strategy in addition to the presence of cognate immunity proteins. Upon binding of the toxic protein by the secretion component, the cell ensures to rapidly deliver the toxin out of the cell where it can execute its damaging effect. However, when lacking the cognate secretion component, the toxin would accumulate within the cell likely leading to cellular damage. In this case, the cell would trigger degradation of the toxin to quickly prevent deleterious outcomes.

**FIGURE 4:**
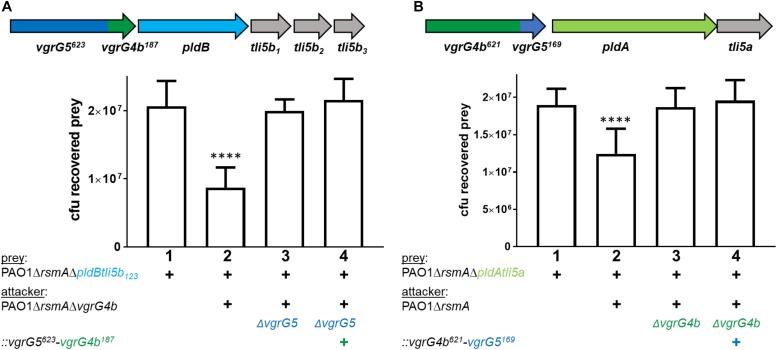
Modifying the VgrG C-terminus abrogates delivery of cognate effectors. The *vgrG* cluster color code is as in Figure 1. The mutant strains used carry the gene sequence for **(A)** *vgrG5^623^-vgrG4b^187^* chimera and **(B)** *vgrG4b^621^-vgrG5^169^* chimera. Note that mutants in **(A)** encode the N terminal 623 amino acids of VgrG5 (blue) and the last C-terminal 187 amino acids of VgrG4b (+, green, Supplementary Figure S4B) and mutants in **(B)** encode the N terminal 621 amino acids of VgrG4b and the last C-terminal 169 amino acids of VgrG5 (+, Supplementary Figure S4C). Bottom panels: bacterial competition experiments and plots of recovered cfu of prey strains as described in Figure 1. Recovery is after contact with the attacker strain that encodes **(A)** native *vgrG5* or the *vgrG5^623^-vgrG4b^187^* chimera (+) or **(B)** native *vgrG4b* or the *vgrG4b^621^-vgrG5^169^* (+). As a positive control for effector-mediated killing, the parental strains were included (lanes 2), while the negative control lacked the cognate *vgrG* (lanes 3). Spots were incubated for 24 h at 25 ^∘^C in a 1:1 ratio. One-Way ANOVA analysis with Dunnett’s multiple comparisons test was conducted on data set obtained from recovered prey on their own with ^*⁣*⁣**^p < 0.0001.

All our results indicate that PldA and PldB are specifically stabilized, and likely chaperoned, by the C-terminal TTR domains of their cognate VgrG proteins. If this were the case, a modified VgrG C-terminal extension domain would jeopardize the cognate effector delivery. We tested this hypothesis and used appropriate *P. aeruginosa* strains producing VgrG4b^621^-VgrG5^169^ or VgrG5^623^-VgrG4b^187^ chimera to challenge prey cells susceptible to PldB (Figure 4A) or PldA (Figure 4B) injection. Note that the susceptibility is conferred by the deletion of appropriate immunity genes, *tli5b*_123_ or *tli5a* in the prey cells. Remarkably, attacker strains with a modified cognate VgrG (Figure 4, lanes 4, respectively) failed to outcompete corresponding susceptible prey cells thus confirming that the C-terminal domain of VgrG is instrumental for effector recognition and delivery.

### Swapping the C-Terminal TTR-Like Domain to Redirect Effectors to the VgrG Tip

**FIGURE 5:**
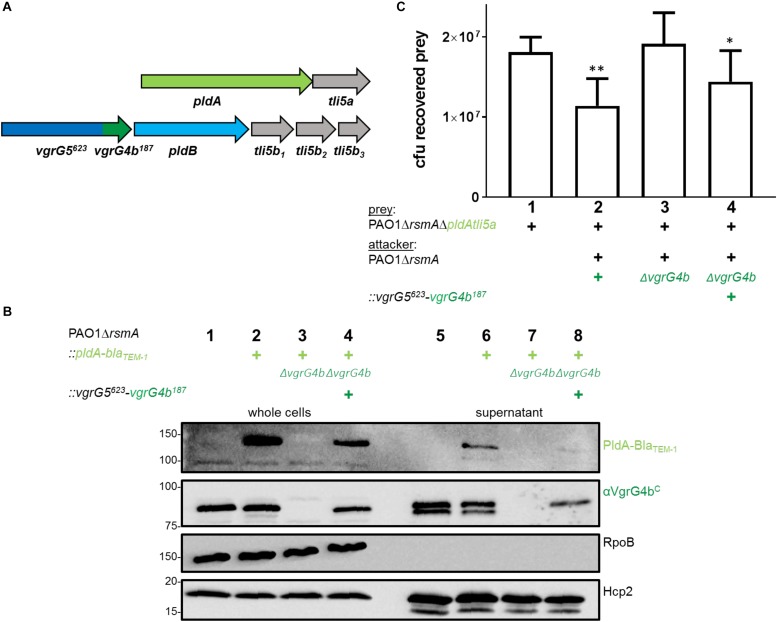
Substitution of the VgrG5 C-terminus with the VgrG4b C-terminus restores PldA delivery. **(A)** Schematic depicting a strain carrying the *vgrG5^623^-vgrG4b^187^* chimeric gene at the native *vgrG5.* The native *vgrG4b* is deleted. **(B)** Representative figure of a western blot from a secretion assay using PAO1Δ*rsmA* or PAO1Δ*rsmA::pldA-bla_TEM–1_* (+) encoding native *vgrG4b* or the *vgrG5^623^-vgrG4b^187^* chimera (+). As a control for PldA-Bla_TEM–1_ secretion, the parental strain was included (lane 6), while the negative control lacked the native *vgrG4b* (lane 7). Antibodies used (from top to bottom) are against Bla_TEM–1_, VgrG4b^C^, RpoB and Hcp2 as indicated on the right. **(C)** Bacterial competition represented by plots of recovered cfu of prey strain PAO1Δ*rsmA*Δ*pldAtli5a::lacZ* after contact with the attacker strain that encodes native *vgrG4b* or the *vgrG5^623^-vgrG4b^187^* chimera (+). As a positive control for PldA-mediated killing, PAO1Δ*rsmA* was included (lane 2), while the negative control lacked *vgrG4b* (lane 3). Spots were incubated for 24 h at 25 ^∘^C in a 1:1 ratio. One-Way ANOVA analysis with Dunnett’s multiple comparisons test was conducted on data set obtained from recovered prey on their own with ^*⁣*⁣**^p < 0.0001.

We then sought to exploit the concept of VgrG C-terminal specificity to force PldA to use a VgrG5 vehicle carrying the VgrG4b C terminus, i.e., a VgrG5^623^-VgrG4b^187^ chimera (Figure 5A). Remarkably, PldA was identified in the supernatant fraction of cells expressing VgrG5^623^-VgrG4b^187^, which was also secreted (Figure 5B, lane 8). We also performed competition assays and showed that cells expressing VgrG5^623^-VgrG4b^187^ display a competitive advantage toward prey cells lacking the *pldAtli5a* effector-immunity locus, which are thus PldA-sensitive (Figure 5C). These results suggest that presence of the C-terminal domain of VgrG4b at the VgrG5 tip is sufficient to adapt VgrG5 for PldA delivery and that it is possible to redirect an effector to a non-cognate VgrG vehicle.

### Interdependent VgrG Secretion Compromises the Swapping Specificity Hypothesis

**FIGURE 6:**
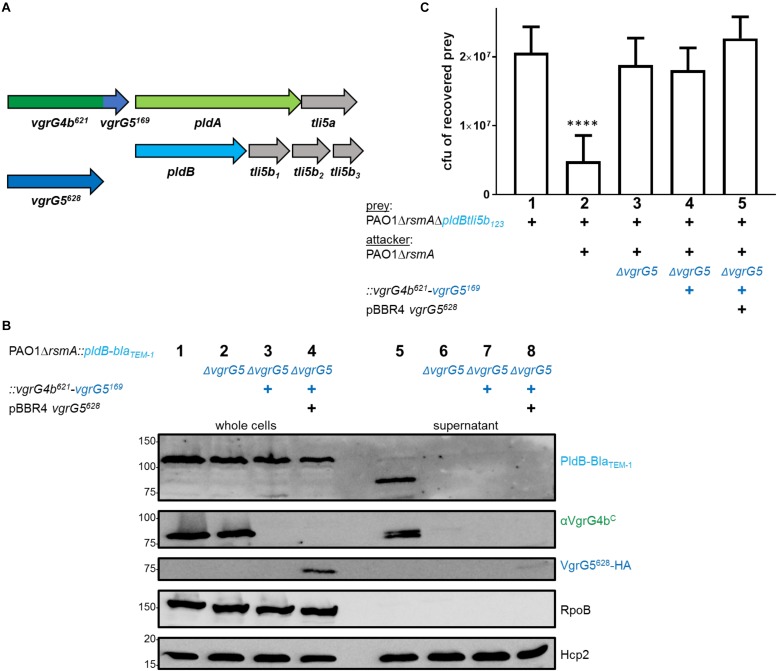
Production of the VgrG5 N-terminus (VgrG5^628^) does not support PldB secretion. **(A)** Schematic depicting a strain carrying the chimeric gene *vgrG4b^629^-vgrG5^169^* at the native *vgrG4b* locus and in absence of the native *vgrG5* gene. A construct encoding a quadruple HA-tagged version of VgrG5^628^ is expressed from *pBBR1-mcs4* (pBBR4-*vgrG5*^628^). **(B)** Representative figure of a western blot of a secretion assay using PAO1Δ*rsmA::pldB-bla_TEM–1_* in presence of the native *vgrG5*, encoding the *vgrG4b^629^-vgrG5^169^* chimera (+) or carrying pBBR4*-vgrG5^628^* (+). Antibodies used (from top to bottom) are against Bla_TEM–1_, VgrG4b^C^, HA, RpoB and Hcp2 as indicated on the right. **(C)** Bacterial competition represented by plot of recovered cfu of prey strain PAO1Δ*rsmA*Δ*pldBtli5b_3_::lacZ* after contact with the attacker strain that encoded native *vgrG5* or the *vgrG4b^629^-vgrG5^169^* chimera (+) or pBBR1*-mcs4 vgrG5*^628^ (+). As a positive control for PldB-mediated killing, PAO1Δ*rsmA* was included (lane 2), while the negative control lacked the native *vgrG5* locus (lane 3). Spots were incubated for 24 h at 25 ^∘^C in a 1:1 ratio. One-Way ANOVA analysis with Dunnett’s multiple comparisons test was conducted on data set obtained from recovered prey on their own with ^*⁣*⁣**^p < 0.0001.

We further aimed to test whether the same concept would hold true to adapt the effector PldB to a non-cognate VgrG vehicle. Indeed, when using the VgrG4b^621^-VgrG5^169^ chimera to specifically bind and deliver PldB (Figure 6A), neither secretion (Figure 6B, lane 7) nor delivery into prey cells (Figure 6C, lane 4) could be observed. One plausible explanation is that VgrG4b secretion is abolished in absence of VgrG5 (Figure 6B, lane 6 and Figure 1B, lane 5). This would further impact secretion of the VgrG4b^621^-VgrG5^169^ chimera as it is expressed in a *vgrG5*-deficient background to avoid cross specificity for the cognate effector PldB. Nevertheless, absence of neither VgrG4b nor VgrG4b^621^-VgrG5^169^ secretion is due to an inactive H2-T6SS, as Hcp2 is efficiently secreted (Figure 1B, lane 5 and Figure 6B, lane 6). This result challenged our concept that an effector can be delivered *via* any VgrG vehicle solely by equipping it with the appropriate C-terminal domain.

**FIGURE 7:**
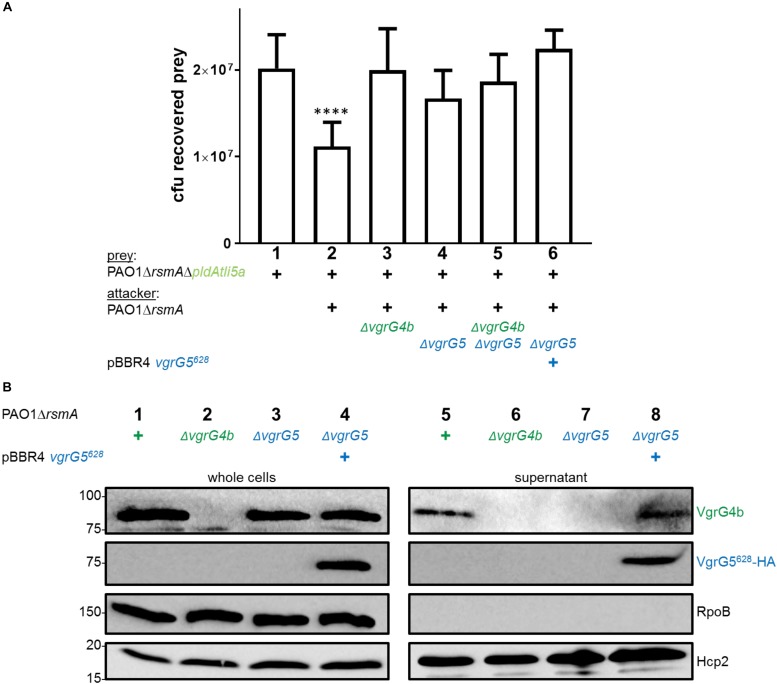
VgrG5 mediates delivery of both PldA and PldB. **(A)** Plots of recovered cfu of prey strains susceptible for PldA delivery upon bacterial competition assays. Prey strains are as described in Figure 1. PAO1Δ*rsmA* attacking strains either lacked (Δ) *vgrG4b*, *vgrG5* or both, or expressed *vgrG5*^628^ *in trans* (+). Spots were incubated for 24 h at 25 ^∘^C in a 1:1 ratio. One-Way ANOVA analysis with Dunnett’s multiple comparisons test was conducted on the data set obtained from recovered prey on their own with ^*⁣*⁣**^p < 0.0001. **(B)** Representative figure of western blot from a secretion assay using PAO1Δ*rsmA.* The absence (Δ) of the native *vgrG5* or *vgrG4b* is shown as well as when expressing an HA-tagged version of *vgrG5*^628^ *in trans* (+). Antibodies (from top to bottom) against the C-terminal portion of VgrG4b (VgrG4b), HA, RpoB and Hcp2 were used as indicated on the right.

Since VgrG4b mediates PldA delivery, we hypothesized that VgrG5 absence would thus in turn affect PldA delivery into prey cells, which appeared to be the case (Figure 7A, lane 4). A straightforward explanation for VgrG4b secretion being dependent on VgrG5 would be that both might be part of a hetero-trimer. Indeed, in both *P. aeruginosa* and *V. cholerae*, it has been suggested that the spike can be made of a hetero-complex of various VgrG proteins (Pukatzki et al., 2007; Hachani et al., 2011). Hence, we investigated whether VgrG4b could be secreted as part of a hetero-trimer with VgrG5. In Figure 1B, middle panel, lane 6, we showed that presence of full length VgrG5 is sufficient to restore VgrG4b secretion. Since trimerization of VgrG proteins to a functional spike is mediated by their gp5/gp27-like domains (Spinola-Amilibia et al., 2016), we hypothesized that expression of solely the VgrG5 N-terminal domain (first 628 amino acids, VgrG5^628^) would suffice to form a functional spike. By performing a secretion assay using a *vgrG5* mutant, we indeed verified that VgrG4b secretion is restored in presence of VgrG5^628^ only (Figure 7B, lane 8). This also confirmed that the C terminus of VgrG5 is not required to support VgrG4b secretion.

Since VgrG4b is secreted in presence of VgrG5^628^, we reasoned that this construct would restore PldA delivery, which was not observed (Figure 7A, lane 6). There are potential explanations for this observation. One would be the existence of an effector delivery hierarchy. For example, VgrG5 homotrimer-dependent delivery of PldB might be initially required for subsequent VgrG4b homotrimer-dependent delivery of PldA. As such, using the truncated VgrG5^628^ would not suffice to trigger VgrG4b-PldA delivery since PldB was not delivered. We tested this hypothesis by monitoring PldA delivery in absence of PldB (Supplementary Figure S4). An attacking strain lacking PldB showed to have the same competitive advantage toward a PldA-lacking strain (lane 5) as the parental strain (lane 2). Hence, the effector hierarchy concept is not fully supported by this observation.

An alternative possibility could be that another T6SS component, such as an adaptor or chaperone, binds the C-terminal domains of VgrG5 and VgrG4b in a VgrG4b-VgrG5 hetero-trimer and thus triggers binding of PldA toward the spike. There is for example evidence suggesting that the chaperone TecT connects the effector TseT to a VgrG4b-VgrG6 hetero-trimer (Burkinshaw et al., 2018). A similar concept could be possible for a VgrG4b-VgrG5-PldA complex. However, the finding that PldA is delivered from a VgrG5^623^-VgrG4b^187^ chimera lacking the C-terminal domain of VgrG5 (Figure 5) is not in full agreement with this hypothesis. Yet it is clear that VgrG5 does not need another VgrG for its secretion and is able to deliver any effector as long as it carries a cognate C-terminal domain, here PldA through the Vgr4b C-terminal domain.

We hypothesized that co-expression of the VgrG5^628^ construct would also facilitate delivery of the VgrG4b^621^-VgrG5^169^ chimera, which in turn would mediate PldB delivery (Figure 6A). However, neither PldB secretion (Figure 6B, lane 8) nor PldB-mediated killing (Figure 6C, lane 5) could be observed. This result might be explained by the lack of recognition of a cognate PAAR protein, which specifically binds the VgrG tip (Shneider et al., 2013). Here, we starkly modified the PAAR recognition site, which might have rendered this tip unrecognizable for its cognate PAAR protein and thus unsuitable for secretion.

**FIGURE 8:**
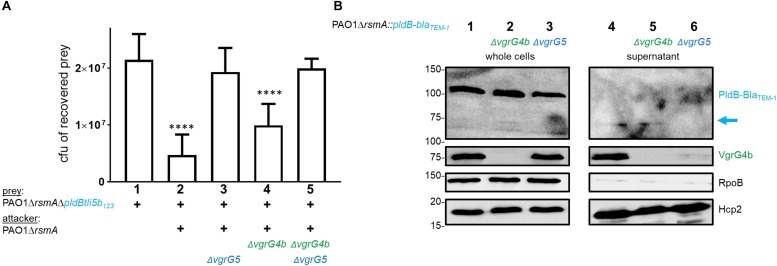
Absence of VgrG4b has no impact on PldB delivery. **(A)** Plots of recovered cfu of prey strains susceptible for PldB delivery upon bacterial competition assays. Prey strains are as described in Figure 1. PAO1Δ*rsmA* attacking strains either lacked (Δ) *vgrG4b*, *vgrG5* or both. Spots were incubated for 24 h at 25^∘^C in a 1:1 ratio. One-Way ANOVA analysis with Dunnett’s multiple comparisons test was conducted on the data set obtained from recovered prey on their own with ^*⁣*⁣**^p < 0.0001. **(B)** Representative figure of a western blot from a secretion assay using PAO1Δ*rsmA::pldB-bla_TEM–1_.* Strains lack (Δ) native *vgrG4b* or *vgrG5*. The blue arrow on the right indicates the secretion product of PldB-Bla_TEM–1_, which is not visible when *vgrG5* is deleted. Antibodies used (from top to bottom) are against Bla_TEM–1_, VgrG4b^C^, RpoB and Hcp2 as indicated on the right.

Due to the impact of VgrG5 on PldA delivery, we finally questioned whether VgrG4b would be involved in PldB delivery (Figure 8). We challenged PldB-sensitive prey strains with attackers deficient for *vgrG4b*, *vgrG5* or both and observed that VgrG4b absence reduced PldB delivery, while significant killing could still be observed (Figure 8A, lane 4). We confirmed these data with a secretion assay demonstrating that PldB is still secreted into the supernatant in VgrG4b absence (Figure 8B, lane 5). From these results we conclude that VgrG5 alone suffices for PldB delivery, while PldA delivery depends on both VgrG4b and VgrG5.

### VgrG4b Can Deliver PldA When Covalently Linked to the Spike

In the previous sections we made clear that a VgrG spike can recognize its cognate effector likely through specific protein-protein interaction. It is also known that many VgrGs, called “evolved” VgrGs display an effector domain fused at their C terminus (Ma et al., 2009; Sana et al., 2015). We wonder whether there is a rationale behind having the effector fused to the VgrG C terminus versus a protein-protein interaction delivery mode, or whether this is only the result of a fortuitous evolutionary process.

**FIGURE 9:**
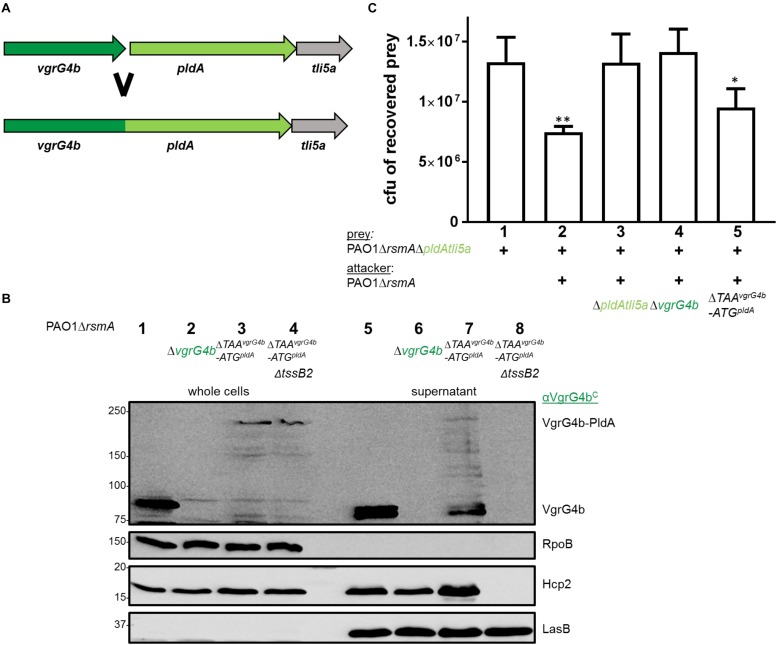
VgrG4b can deliver PldA when fused to its C-terminus. **(A)** The STOP codon of *vgrG4b*, the intergenic region and START-codon of *pldA* were deleted resulting in a single chimeric gene product. **(B)** Representative figure of a western blot from a secretion assay using PAO1Δ*rsmA* with an active or inactive H2-T6SS (Δ*tssB2*) encoding VgrG4b-PldA (Δ*TAA^vgrG4b^-ATG^pldA^*). As a positive control for VgrG4b secretion, PAO1Δ*rsmA* (lane 1) was included, while the negative control lacked *vgrG4b* (lane 2). Antibodies used (from top to bottom) are against VgrG4b^C^, RpoB, Hcp2 and LasB, a T2SS substrate (Olson and Ohman, 1992) acting as a loading control for the supernatant, as indicated on the right. **(C)** Bacterial competition represented by plots of recovered cfu of prey strain PAO1Δ*rsmA*Δ*pldAtli5a*:: *lacZ* after contact with the attacker strain encoding WT PldA, WT VgrG4b or the fusion VgrG4b-PldA (Δ*TAA^vgrG4b^-ATG^pldA^*) as indicated. As a positive control for PldA-mediated killing, PAO1Δ*rsmA* was included (lane 2) while the negative controls lacked *pldAtli5a* (lane 3) or *vgrG4b* (lane 4). Spots were incubated for 24 h at 25 ^∘^C in a 1:1 ratio. One-Way ANOVA analysis with Dunnett’s multiple comparisons test was conducted on the data set obtained from the recovered prey on its own with ^∗^*p* < 0.05 and ^∗∗^*p* < 0.01.

We demonstrated that PldA is delivered as a cargo effector dependent on VgrG4b and here assessed whether delivery is compromised if PldA is covalently linked to the VgrG4b C terminus. Hence, we deleted the STOP codon of *vgrG4b*, the intergenic region and the START codon of *pldA* (Figure 9A). Expression of this gene would thus lead to production of one single polypeptide consisting of VgrG4b and PldA, which we could readily detect using western blot analysis (Figure 9B, lane 3). The same product was detected in the supernatant fraction of H2-T6SS active strains (Figure 9B, lane 7), but not of H2-T6SS inactive strains (Figure 9B, lane 8) suggesting that secretion of the VgrG4b-PldA fusion remains H2-T6SS-dependent. Note, that additional bands most likely representing VgrG4b degradation products can be detected in the supernatant fraction but to a lesser amount as in the whole cells. This suggests that the artificially evolved VgrG4b-PldA protein becomes prone for proteolysis once secreted into the environment.

We further investigated whether such a fusion could be injected into bacterial prey cells. We challenged PldA-sensitive preys against *P. aeruginosa* strains expressing the VgrG4b-PldA fusion (Figure 9C, lane 5) and observed that they are outcompeted to a similar extent as when in competition with the parental strain (Figure 9C, lane 2). This led us to conclude that the VgrG4b-PldA fusion protein can be delivered by *P. aeruginosa* both into the extracellular milieu and into neighboring bacteria.

### The H2-T6SS Could Be a Core System for the Delivery of Remote VgrG Spikes

**FIGURE 10:**
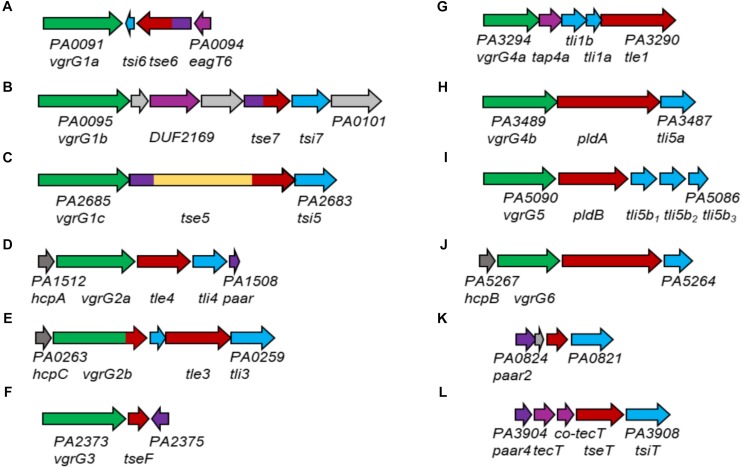
Genomic loci containing *vgrG* or *paar* genes of *P. aeruginosa* PAO1. *vgrG* genes are shown in green, effector genes in red, cognate immunities in cyan, *hcp* genes in dark gr**a**y, *paar* genes in purple, *tap* genes in magenta and genes of unknown function in gr**a**y. For all examples, the PA numbers of the two outer genes are shown. Effector genes close to their (putative) cognate vgrG or PAAR genes are the following: **(A)** tse6; **(B)** tse7; **(C)** tse5; **(D)** tle4; **(E)** tle3; **(F)** tseF; **(G)** tle1; **(H)** pldA; **(I)** pldB; **(J)** PA5265; **(K)** PA0822; **(L)** tseT.

We previously showed that *P. aeruginosa* uses its H1-T6SS to deliver at least three different VgrG-dependent antibacterial effectors into target prey cells, namely Tse5, Tse6 and Tse7 (Hachani et al., 2014; Pissaridou et al., 2018). All of these effectors are evolved PAARs that interact with their cognate VgrGs, VgrG1c, VgrG1a and VgrG1b (Figures 10A–C), respectively, *via* their N-terminal PAAR domains (Shneider et al., 2013; Whitney et al., 2015). In case of the H3-T6SS, information about associated effectors is scarce. In the H3-T6SS cluster, *vgrG3* as well as the effector *tseF* are encoded (Figure 10F) and it is proposed that TseF secretion is H3-T6SS-dependent (Lin et al., 2017). Although not experimentally demonstrated, it is likely that considering the genetic linkage, TseF delivery might also be VgrG3-dependent. Previous reports have also suggested that PldB delivery is H3-T6SS-dependent (Jiang et al., 2014), however, here we show that this effector is secreted in a H2-T6SS-dependent fashion and requires the cognate VgrG5.

There is experimental evidence that the H2-T6SS is responsible for delivery of VgrG4b (Allsopp et al., 2017), VgrG5 (this study), VgrG2a and VgrG2b (Sana et al., 2015). We here provide functional evidence, that VgrG4b specifically delivers PldA (Figure 10H) and VgrG5 delivers PldB (Figure 10I). VgrG2a and VgrG2b are genetically linked to the effector proteins Tle4 (Figure 10D) and Tle3 (Figure 10E; Jiang et al., 2016), however, their functional links require further investigations. Additionally, VgrG4a, which is encoded on a separate satellite *vgrG* island, is likely affiliated with the H2-T6SS due to a sequence identity of 97% with VgrG4b within the N-terminal gp5/gp27-like domains (Allsopp et al., 2017). Downstream of *vgrG4a*, another effector, Tle1, is encoded (Figure 10G), and because of the genetic linkage, most likely delivered in a VgrG4a-dependent manner. VgrG6, encoded on yet another satellite *vgrG* island, is genetically linked to *hcpB* (Figure 10J). This might be an indication for its association with the H2-T6SS (Burkinshaw et al., 2018) because the HcpB amino acids sequence is 100 % identical to HcpA and HcpC (Jones et al., 2013), which are genetically linked to genes encoding H2-T6SS spike proteins VgrG2a (Figure 10D) and VgrG2b (Figure 10E), respectively. Additionally, there is a putative effector gene linked to *vgrG6* whose role has not been studied yet. A remote *paar* cluster (Figure 10L) has also been connected to the H2-T6SS, whose gene product PAAR4 mediates delivery of the effector TseT in a TecT, Co-TecT-, VgrG4b- and VgrG6-dependent fashion (Burkinshaw et al., 2018). PAAR2, encoded on yet another orphan island (Figure 10K), was shown to be able to functionally replace PAAR4 (Burkinshaw et al., 2018), hence its affiliation with the H2-T6SS is also implicated.

Combining these data and observations, we propose that *P. aeruginosa* uses its H2-T6SS machinery to deliver a multitude of different spikes decorated with various effectors. Interestingly, five (PldA, PldB, Tle3, Tle4 and Tle1) out of seven postulated H2-T6SS-dependent effectors are lipases that have been either experimentally proven (Russell et al., 2013; Jiang et al., 2014, 2016) or proposed due to the presence of the lipase-specific DUF2235 domain. The affiliation of at least five lipases, even though with different substrate specificities (Russell et al., 2013), with one T6SS machinery begs the question of why there would be such effector redundancy. In any case, with such a broad spike repertoire, *P. aeruginosa* has many options for loading its H2-T6SS weapon in order to confer a competitive advantage in a polymicrobial environment. This also mitigates the presumption of the H1-T6SS being the major antibacterial T6SS of *P. aeruginosa* (Hachani et al., 2014; Allsopp et al., 2017) while the H2-T6SS provides *P. aeruginosa* with the versatility to fire various effectors into competing prey cells.

## Conclusion

The T6SS is a potent bacterial weapon that delivers an arsenal of toxins into eukaryotic and prokaryotic prey cells. All *P. aeruginosa* strains sequenced so far carry three distinct T6SSs, namely H1-, H2- and H3-T6SS, which could act in concert to inject a lethal cocktail of enzymes targeting essential functions in living organisms. Among these are phospholipases, and notably PldA and PldB, which are described in this study and which are considered as trans-kingdom effectors since they challenge the survival of eukaryotic cells as much as bacterial cells. The effector delivery strategy of the T6SS has been shown to be quite variable, and one mode we described here involves an exquisite recognition specificity between the toxin and the cognate T6SS spike, known as a VgrG trimer. The effector specificity lies within the C-terminal domain of a VgrG and we showed that such region in VgrG4b and VgrG5 is a TTR-like domain providing chaperone activity on PldA and PldB, respectively, which is instrumental for efficient secretion of these effectors (Supplementary Figure S5). PldA and PldB are encoded remotely from other T6SS core genes but are genetically linked to their cognate VgrG, supporting the concept of *vgrG* islands. As such, the *pldA/vgrG4b* or *pldB/vgrG5* genes, remotely and spread on the entire chromosome, exclusively carry the genes needed to load the T6SS spike (e.g., VgrG/effector or VgrG/Effector/Adaptor) at the tip of the T6SS nanomachine. It thus remains quite elusive on which T6SS core system each of the spike is loaded. Here we showed that both VgrG4b/PldA and VgrG5/PldB are delivered in an H2-T6SS-dependent manner and propose that most *vgrG* island-encoded effectors might actually use the H2-T6SS rather than the H1- or H3-T6SS. It is likely that fitting of the VgrG protein onto the T6SS machine would require specific interactions with the Hcp tube, as was shown recently (Renault et al., 2018). It is thus likely that acquisition of single effectors from other bacterial species by horizontal gene transfer would have to obey a number of rules before they can be fitted on and fired by endogenous T6SSs.

## Author Contributions

AF and SW conceived the study, participated in its design and coordination and wrote the manuscript. TW engineered the *P. aeruginosa* prey strains and optimized the bacterial killing assay conditions. SW performed most of the experiments and SF contributed to additional experiments. All authors discussed, reviewed and approved the final manuscript.

## Conflict of Interest Statement

The authors declare that the research was conducted in the absence of any commercial or financial relationships that could be construed as a potential conflict of interest.

## References

[B1] Allsopp L. P. Wood T. E. Howard S. A. Maggiorelli F. Nolan L. M. Wettstadt S. (2017). RsmA and AmrZ orchestrate the assembly of all three type VI secretion systems in *Pseudomonas aeruginosa*. *Proc. Nat. Acad. Sci. U. S. A.* 114 7707–7712. 10.1073/pnas.1700286114 28673999 PMC5530658

[B2] Basler M. Ho B. T. Mekalanos J. J. (2013). Tit-for-tat: type VI secretion system counterattack during bacterial cell-cell interactions. *Cell* 152 884–894. 10.1016/j.cell.2013.01.042 23415234 PMC3616380

[B3] Becher A. Schweizer H. P. (2000). Integration-proficient *Pseudomonas aeruginosa* vectors for isolation of single-copy chromosomal lacZ and lux gene fusions. *Biotechniques* 29 952. 10.2144/00295bm04 11084852

[B4] Bingle L. E. Bailey C. M. Pallen M. J. (2008). Type VI secretion: a beginner’s guide. *Curr. Opin. Microbiol.* 11 3–8. 10.1016/j.mib.2008.01.006 18289922

[B5] Birtalan S. C. Phillips R. M. Ghosh P. (2002). Three-dimensional secretion signals in chaperone-effector complexes of bacterial pathogens. *Mol. cell* 9 971–980. 10.1016/s1097-2765(02)00529-4 12049734

[B6] Bondage D. D. Lin J. S. Ma L. S. Kuo C. H. Lai E. M. (2016). VgrG C terminus confers the type VI effector transport specificity and is required for binding with PAAR and adaptor-effector complex. *Proc. Nat. Acad. Sci. U. S. A.* 113 E3931–E3940. 10.1073/pnas.1600428113 27313214 PMC4941472

[B7] Boulant T. Boudehen Y. M. Filloux A. Plesiat P. Naas T. Dortet L. (2018). Higher prevalence of PldA, a *Pseudomonas aeruginosa* trans-kingdom H2-Type VI secretion system effector, in clinical isolates responsible for acute infections and in multidrug resistant strains. *Front. Microbiol.* 9:2578. 10.3389/fmicb.2018.02578 30420847 PMC6215852

[B8] Browning C. Shneider M. M. Bowman V. D. Schwarzer D. Leiman P. G. (2012). Phage pierces the host cell membrane with the iron-loaded spike. *Structure* 20 326–339. 10.1016/j.str.2011.12.009 22325780

[B9] Brunet Y. R. Henin J. Celia H. Cascales E. (2014). Type VI secretion and bacteriophage tail tubes share a common assembly pathway. *EMBO Rep.* 15 315–321. 10.1002/embr.201337936 24488256 PMC3989698

[B10] Brunet Y. R. Zoued A. Boyer F. Douzi B. Cascales E. (2015). The type VI secretion TssEFGK-VgrG phage-like baseplate is recruited to the TssJLM membrane complex via multiple contacts and serves as assembly platform for tail tube/sheath polymerization. *PLoS Genet.* 11:e1005545. 10.1371/journal.pgen.1005545 26460929 PMC4604203

[B11] Burkinshaw B. J. Liang X. Wong M. Le A. N. H. Lam L. Dong T. G. (2018). A type VI secretion system effector delivery mechanism dependent on PAAR and a chaperone-co-chaperone complex. *Nat. Microbiol.* 3 632–640. 10.1038/s41564-018-0144-4 29632369

[B12] Diniz J. A. Coulthurst S. J. (2015). Intra-species competition in *Serratia marcescens* is mediated by type VI secretion Rhs effectors and a conserved effector-associated accessory protein. *J. Bacteriol.* 197 2350–2360. 10.1128/jb.00199-15 25939831 PMC4524185

[B13] Durand E. Nguyen V. S. Zoued A. Logger L. Pehau-Arnaudet G. Aschtgen M. S. (2015). Biogenesis and structure of a type VI secretion membrane core complex. *Nature* 523 555–560. 10.1038/nature14667 26200339

[B14] Durand E. Zoued A. Spinelli S. Watson P. J. Aschtgen M. S. Journet L. (2012). Structural characterization and oligomerization of the TssL protein, a component shared by bacterial type VI and type IVb secretion systems. *J. Biol. Chem.* 287 14157–14168. 10.1074/jbc.M111.338731 22371492 PMC3340138

[B15] Figurski D. H. Helinski D. R. (1979). Replication of an origin-containing derivative of plasmid RK2 dependent on a plasmid function provided in trans. *Proc. Nat. Acad. Sci.U. S. A.* 76 1648–1652. 10.1073/pnas.76.4.1648 377280 PMC383447

[B16] Flaugnatti N. Le T. T. Canaan S. Aschtgen M. S. Nguyen V. S. Blangy S. (2015). A phospholipase a anti-bacterial T6SS effector interacts directly with the C-terminal domain of the VgrG spike protein for delivery. *Mol. Microbiol.* 99 1099–1118. 10.1111/mmi.13292 26714038

[B17] Hachani A. Allsopp L. P. Oduko Y. Filloux A. (2014). The VgrG proteins are “á la carte” delivery systems for bacterial type VI effectors. *J. Biol. Chem.* 289 17872–17884. 10.1074/jbc.M114.563429 24794869 PMC4067218

[B18] Hachani A. Lossi N. S. Filloux A. (2013). A visual assay to monitor T6SS-mediated bacterial competition. *J. Vis. Exp.* 73:e50103. 10.3791/50103 PMC3639552 23542679

[B19] Hachani A. Lossi N. S. Hamilton A. Jones C. Bleves S. Albesa-Jove D. (2011). Type VI secretion system in *Pseudomonas aeruginosa*: secretion and multimerization of VgrG proteins. *J. Biol. Chem.* 286 12317–12327. 10.1074/jbc.M110.193045 21325275 PMC3069435

[B20] Herrero M. de Lorenzo V. Timmis K. N. (1990). Transposon vectors containing non-antibiotic resistance selection markers for cloning and stable chromosomal insertion of foreign genes in gram-negative bacteria. *J. Bacteriol.* 172 6557–6567. 10.1128/jb.172.11.6557-6567.1990 2172216 PMC526845

[B21] Jiang F. Wang X. Wang B. Chen L. Zhao Z. Waterfield N. R. (2016). The *Pseudomonas aeruginosa* type VI secretion PGAP1-like effector induces host autophagy by activating endoplasmic reticulum stress. *Cell Rep.* 16 1502–1509. 10.1016/j.celrep.2016.07.012 27477276

[B22] Jiang F. Waterfield N. R. Yang J. Yang G. Jin Q. (2014). A *Pseudomonas aeruginosa* type VI secretion phospholipase D effector targets both prokaryotic and eukaryotic cells. *Cell Host Microbe* 15 600–610. 10.1016/j.chom.2014.04.010 24832454

[B23] Jones C. Hachani A. Manoli E. Filloux A. (2013). An *rhs* gene linked to the second type VI secretion cluster is a feature of the *Pseudomonas aeruginosa* strain PA14. *J. Bacteriol.* 196 800–810. 10.1128/JB.00863-13 24317402 PMC3911176

[B24] Kanamaru S. Leiman P. G. Kostyuchenko V. A. Chipman P. R. Mesyanzhinov V. V. Arisaka F. (2002). Structure of the cell-puncturing device of bacteriophage T4. *Nature* 415 553–557. 10.1038/415553a 11823865

[B25] Kaniga K. Delor I. Cornelis G. R. (1991). A wide-host-range suicide vector for improving reverse genetics in gram-negative bacteria: inactivation of the blaA gene of Yersinia enterocolitica. *Gene* 109 137–141. 10.1016/0378-1119(91)90599-7 1756974

[B26] Kovach M. E. Elzer P. H. Hill D. S. Robertson G. T. Farris M. A. Roop R. M. II (1995). Four new derivatives of the broad-host-range cloning vector pBBR1MCS, carrying different antibiotic-resistance cassettes. *Gene* 166 175–176. 10.1016/0378-1119(95)00584-1 8529885

[B27] Leiman P. G. Basler M. Ramagopal U. A. Bonanno J. B. Sauder J. M. Pukatzki S. (2009). Type VI secretion apparatus and phage tail-associated protein complexes share a common evolutionary origin. *Proc. Nat. Acad. Sci. U. S. A.* 106 4154–4159. 10.1073/pnas.0813360106 19251641 PMC2657435

[B28] Liang X. Moore R. Wilton M. Wong M. J. Lam L. Dong T. G. (2015). ). Identification of divergent type VI secretion effectors using a conserved chaperone domain. *Proc. Nat. Acad. Sci. U. S. A* 112 9106–9111. 10.1073/pnas.1505317112 26150500 PMC4517263

[B29] Lin J. Zhang W. Cheng J. Yang X. Zhu K. Wang Y. (2017). A *Pseudomonas* T6SS effector recruits PQS-containing outer membrane vesicles for iron acquisition. *Nat. Commun.* 8:14888. 10.1038/ncomms14888 28348410 PMC5379069

[B30] Ma A. T. McAuley S. Pukatzki S. Mekalanos J. J. (2009). Translocation of a *Vibrio cholerae* type VI secretion effector requires bacterial endocytosis by host cells. *Cell Host Microbe* 5 234–243. 10.1016/j.chom.2009.02.005 19286133 PMC3142922

[B31] Ma J. Pan Z. Huang J. Sun M. Lu C. Yao H. (2017). The Hcp proteins fused with diverse extended-toxin domains represent a novel pattern of antibacterial effectors in type VI secretion systems. *Virulence* 8 1189–1202. 10.1080/21505594.2017.1279374 28060574 PMC5711352

[B32] Ma L. S. Hachani A. Lin J. S. Filloux A. Lai E. M. (2014). *Agrobacterium tumefaciens* deploys a superfamily of type VI secretion DNase effectors as weapons for interbacterial competition in planta. *Cell Host Microbe* 16 94–104. 10.1016/j.chom.2014.06.002 24981331 PMC4096383

[B33] MacIntyre D. L. Miyata S. T. Kitaoka M. Pukatzki S. (2010). The *Vibrio cholerae* type VI secretion system displays antimicrobial properties. *Proc. Nat. Acad. Sci. U. S. A.* 107 19520–19524. 10.1073/pnas.1012931107 20974937 PMC2984155

[B34] Olson J. C. Ohman D. E. (1992). Efficient production and processing of elastase and LasA by *Pseudomonas aeruginosa* require zinc and calcium ions. *J. Bacteriol.* 174 4140–4147. 10.1128/jb.174.12.4140-4147.1992 1597429 PMC206126

[B35] Pissaridou P. Allsopp L. P. Wettstadt S. Howard S. A. Mavridou D. A. I. Filloux A. (2018). The *Pseudomonas aeruginosa* T6SS-VgrG1b spike is topped by a PAAR protein eliciting DNA damage to bacterial competitors. *Proc. Nat. Acad. Sci. U. S. A.* 115 12519–12524. 10.1073/pnas.1814181115 30455305 PMC6298103

[B36] Planamente S. Salih O. Manoli E. Albesa-Jove D. Freemont P. S. Filloux A. (2016). TssA forms a gp6-like ring attached to the type VI secretion sheath. *EMBO J.* 35 1613–1627. 10.15252/embj.201694024 27288401 PMC4969574

[B37] Pukatzki S. Ma A. T. Revel A. T. Sturtevant D. Mekalanos J. J. (2007). Type VI secretion system translocates a phage tail spike-like protein into target cells where it cross-links actin. *Proc. Nat. Acad. Sci. U. S. A.* 104 15508–15513. 10.1073/pnas.0706532104 17873062 PMC2000545

[B38] Pukatzki S. Ma A. T. Sturtevant D. Krastins B. Sarracino D. Nelson W. C. (2006). Identification of a conserved bacterial protein secretion system in *Vibrio cholerae* using the dictyostelium host model system. *Proc. Nat. Acad. Sci. U. S. A.* 103 1528–1533. 10.1073/pnas.0510322103 16432199 PMC1345711

[B39] Quentin D. Ahmad S. Shanthamoorthy P. Mougous J. D. Whitney J. C. Raunser S. (2018). Mechanism of loading and translocation of type VI secretion system effector Tse6. *Nat. Microbiol.* 3 1142–1152. 10.1038/s41564-018-0238-z 30177742 PMC6488228

[B40] Renault M. G. Zamarreno Beas J. Douzi B. Chabalier M. Zoued A. Brunet Y. R. (2018). The gp27-like Hub of VgrG Serves as Adaptor to Promote Hcp Tube Assembly. *J. Mol. Biol.* 430(18 Pt B), 3143–3156. 10.1016/j.jmb.2018.07.018 30031895

[B41] Russell A. B. Hood R. D. Bui N. K. LeRoux M. Vollmer W. Mougous J. D. (2011). Type VI secretion delivers bacteriolytic effectors to target cells. *Nature* 475 343–347. 10.1038/nature10244 21776080 PMC3146020

[B42] Russell A. B. LeRoux M. Hathazi K. Agnello D. M. Ishikawa T. Wiggins P. A. (2013). Diverse type VI secretion phospholipases are functionally plastic antibacterial effectors. *Nature* 496 508–512. 10.1038/nature12074 23552891 PMC3652678

[B43] Sana T. G. Baumann C. Merdes A. Soscia C. Rattei T. Hachani A. (2015). Internalization of Pseudomonas aeruginosa strain PAO1 into epithelial cells is promoted by interaction of a T6SS effector with the microtubule network. *MBio* 6:e00712. 10.1128/mBio.00712-15 26037124 PMC4453011

[B44] Schmidt-Eisenlohr H. Domke N. Baron C. (1999). TraC of IncN plasmid pKM101 associates with membranes and extracellular high-molecular-weight structures in *Escherichia coli*. *J Bacteriol* 181 5563–5571. 10482495 10.1128/jb.181.18.5563-5571.1999 PMC94074

[B45] Shneider M. M. Buth S. A. Ho B. T. Basler M. Mekalanos J. J. Leiman P. G. (2013). PAAR-repeat proteins sharpen and diversify the type VI secretion system spike. *Nature* 500 350–353. 10.1038/nature12453 23925114 PMC3792578

[B46] Silverman J. M. Agnello D. M. Zheng H. Andrews B. T. Li M. Catalano C. E. (2013). Haemolysin coregulated protein is an exported receptor and chaperone of type VI secretion substrates. *Mol. Cell* 51 584–593. 10.1016/j.molcel.2013.07.025 23954347 PMC3844553

[B47] Spinola-Amilibia M. Davo-Siguero I. Ruiz F. M. Santillana E. Medrano F. J. Romero A. (2016). The structure of VgrG1 from *Pseudomonas aeruginosa*, the needle tip of the bacterial type VI secretion system. *Acta. Crystallogr. D Struct. Biol.* 72 22–33. 10.1107/S2059798315021142 26894531

[B48] Sutherland M. C. Nguyen T. L. Tseng V. Vogel J. P. (2012). The *Legionella* IcmSW complex directly interacts with DotL to mediate translocation of adaptor-dependent substrates. *PLoS Pathog.* 8:e1002910. 10.1371/journal.ppat.1002910 23028312 PMC3441705

[B49] Unterweger D. Kostiuk B. Otjengerdes R. Wilton A. Diaz-Satizabal L. Pukatzki S. (2015). Chimeric adaptor proteins translocate diverse type VI secretion system effectors in *Vibrio cholerae*. *EMBO J.* 34 2198–2210. 10.15252/embj.201591163 26194724 PMC4557670

[B50] Vasseur P. Vallet-Gely I. Soscia C. Genin S. Filloux A. (2005). The pel genes of the *Pseudomonas aeruginosa* PAK strain are involved at early and late stages of biofilm formation. *Microbiology* 151 985–997. 10.1099/mic.0.27410-0 15758243

[B51] Whitney J. C. Beck C. M. Goo Y. A. Russell A. B. Harding B. N. De Leon J. A. (2014). Genetically distinct pathways guide effector export through the type VI secretion system. *Mol. Microbiol.* 92 529–542. 10.1111/mmi.12571 24589350 PMC4049467

[B52] Whitney J. C. Quentin D. Sawai S. LeRoux M. Harding B. N. Ledvina H. E. (2015). An interbacterial NAD(P)(+) glycohydrolase toxin requires elongation factor Tu for delivery to target cells. *Cell* 163 607–619. 10.1016/j.cell.2015.09.027 26456113 PMC4624332

[B53] Zhao Z. Sagulenko E. Ding Z. Christie P. J. (2001). Activities of virE1 and the VirE1 secretion chaperone in export of the multifunctional VirE2 effector via an *Agrobacterium* type IV secretion pathway. *J. Bacteriol.* 183 3855–3865. 10.1128/jb.183.13.3855-3865.2001 11395448 PMC95267

