# Supplementary material for: Delivery of the Pseudomonas aeruginosa Phospholipase Effectors PldA and PldB in a VgrG- and H2-T6SS-Dependent Manner
Source: Front Microbiol. 2019 Jul 31;10:1718. doi: 10.3389/fmicb.2019.01718 (PMC6684961; doi:10.3389/fmicb.2019.01718)
Supplement: Supplementary file 1 [file Data_Sheet_1.PDF]

Supplementary Material

Delivery of the *Pseudomonas aeruginosa* phospholipase effectors PldA and PldB in a VgrG- and H2-T6SS-dependent manner

Sarah Wettstadt[1,2], Thomas E. Wood[1,3], Selina Fecht[1] and Alain Filloux[1,]*

*** Correspondence:**

Alain Filloux

a.filloux@imperial.ac.uk

Supplementary Figures

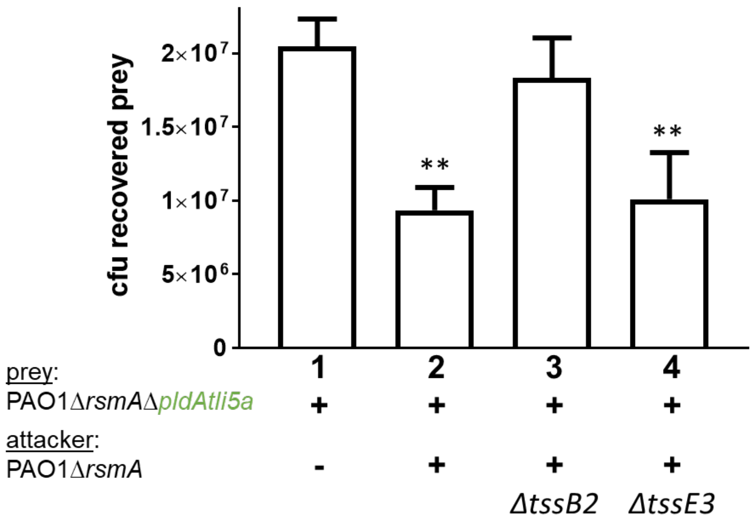

Supplementary Figure S1. PldA delivery is H2-T6SS dependent. Plot of recovered cfu of prey strain PAO1Δ*rsmA*Δ*tlei5a::lacZ* after contact with the attacker strain that encodes a non-functional H2 (*ΔtssB2*)- or H3-T6SS (Δ*tssE3*). As a positive control for PldA-mediated killing, PAO1Δ*rsmA* was included (lane 2). Spots were incubated for 24 h at 25 °C in a 1:1 ratio. One-Way ANOVA analysis with Dunnett's multiple comparisons test was conducted on data set obtained from recovered prey on their own with ** $p<0.01$.

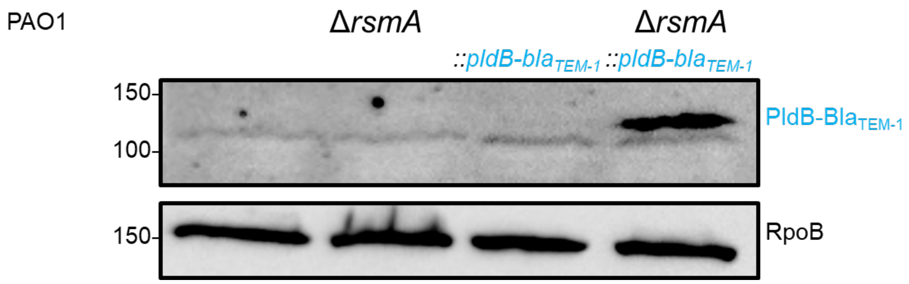

Supplementary Figure S2. PldB is produced in absence of RsmA. Representative figure of a western blot from whole cell lysates derived from PAO1 after 8h growth at 25 °C. Strains carried a clean deletion of rsmA (ΔrsmA) in absence (-) or presence (+) of BlaTEM-1-tagged versions of PldB. Antibodies used (from top to bottom) are against BlaTEM-1 and RpoB as indicated on the right.

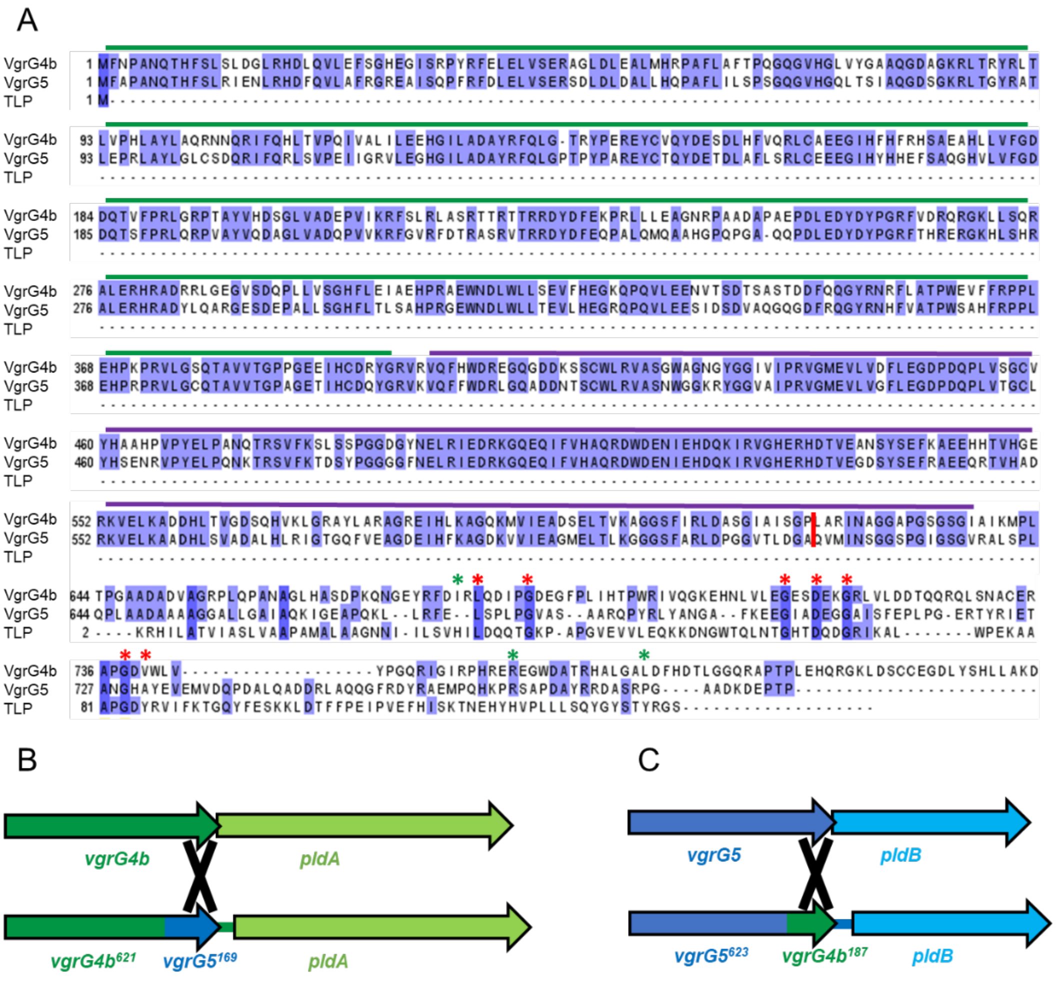

Supplementary Figure S3. A: Multiple sequence alignment between VgrG4b and VgrG5 and the transthyretin-like protein (TLP) from *Salmonella dublin*. Highlighted with green lines are the predicted gp27-like domains and with purple lines the predicted gp5-like domains. Green asterisks correspond to the conserved catalytic residues within the TLP. Note, that these amino acids differ in VgrG4b and VgrG5. The red asterisks highlight the conserved residues of a TTR-motif also present in the C-termini of VgrG4b and VgrG5. The red line indicates the location of the swap of the C-termini (see C and D). B: WT *vgrG4b* (green) is followed by *pldA* (bright green). For this mutant, the gene sequence corresponding to the C-terminus of *vgrG5*^{169} (blue) was used to substitute the gene sequence corresponding to the C-terminus of *vgrG4b*, while 30 bp upstream of the START codon of *pldA* was unchanged. C: WT *vgrG5* (blue) is followed by *pldB* (cyan). For this mutant, the gene sequence corresponding to the C-terminus of *vgrG4b*^{187} was used to substitute the gene sequence corresponding to the C-terminus of *vgrG5*, while 30 bp upstream of the START codon of *pldB* was unchanged.

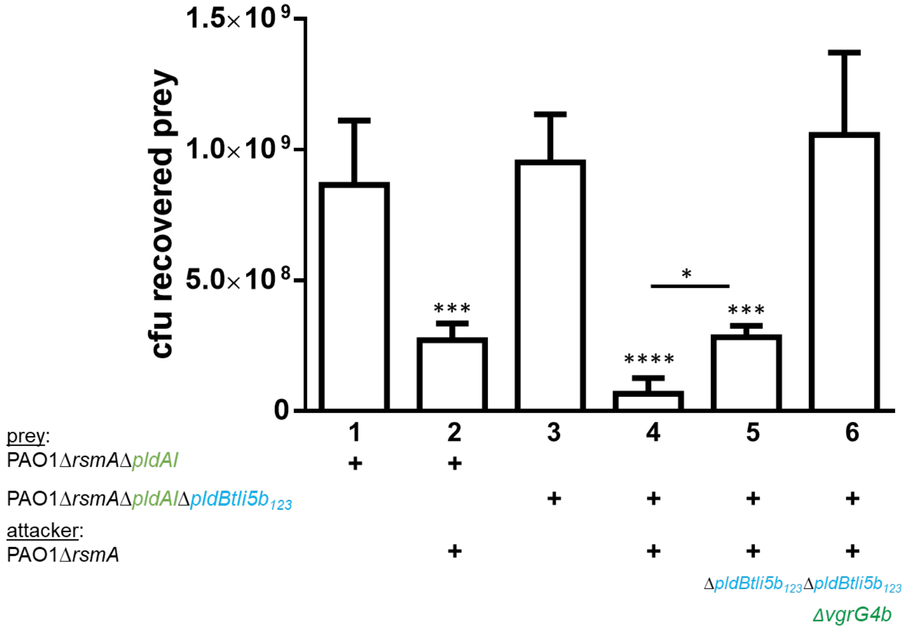

Supplementary Figure S4. PldA delivery is not PldB-dependent. Plot of recovered cfu of prey strain PAO1Δ*rsmA*Δ*pldAI::lacZ* (lanes 1 and 2) or PAO1Δ*rsmA*Δ*pldAI*Δ*pldBtlei*$_{123}$*::lacZ* (lanes 3 to 6) after contact with the attacker strain that lacks *pldBtlei*$_{123}$ (Δ). As a positive control for PldA-mediated killing, PAO1Δ*rsmA* was included (lane 2). Spots were incubated for 24 h at 25 °C in a 1:1 ratio. One-Way ANOVA analysis with Dunnett's multiple comparisons test was conducted on data set obtained from recovered prey on their own with **** $p<0.0001$.

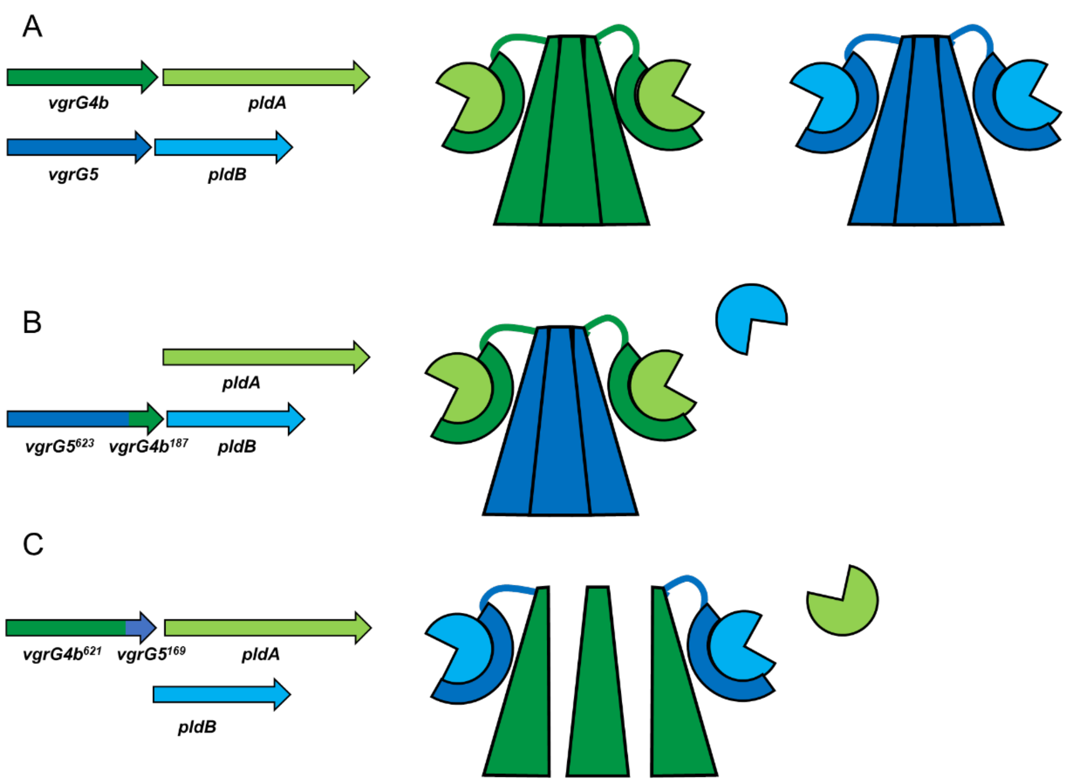

Supplementary Figure S5. The C-terminal domains of VgrG4b and VgrG5 specifically bind and stabilize their cognate effectors prior to their delivery. A: In *P. aeruginosa* WT, VgrG4b (green) and VgrG5 (blue) form trimers that are secreted. PldA (bright green) and PldB (cyan) interact with the C-terminal domains of their cognate VgrGs, VgrG4b and VgrG5, respectively. B: To connect PldA with VgrG5 as a vehicle, the C-terminus of VgrG5 was substituted with the C-terminus of VgrG4b in a *vgrG4b* deficient background. The VgrG5^{623}-VgrG4b^{187} chimera forms a functional tip that recruits PldA, but not PldB, and delivers the cognate effector. C: To connect PldB with VgrG4b as a vehicle, the C-terminus of VgrG4b was substituted with the C-terminus of VgrG5 in a *vgrG5* deficient background. The VgrG4b^{621}-VgrG5^{169} chimera does not form a functional tip but interacts with PldB and not with PldA. No effector is delivered with this construct.
